# Comparative analysis of proteomic adaptations in *Enterococcus faecalis* and *Enterococcus faecium* after long term bile acid exposure

**DOI:** 10.1186/s12866-024-03253-0

**Published:** 2024-04-03

**Authors:** Annika Dreyer, Christof Lenz, Uwe Groß, Wolfgang Bohne, Andreas Erich Zautner

**Affiliations:** 1 Institute for Medical Microbiology and Virology, University Medical Center Göttingen, Göttingen, Germany; 2 Bioanalytical Mass Spectrometry Group, Max Planck Institute for Multidisciplinary Sciences, Göttingen, Germany; 3 Department of Clinical Chemistry, University Medical Center Göttingen, Göttingen, Germany; 4 Institute of Medical Microbiology and Hospital Hygiene, Medical Faculty, Otto-von-Guericke University Magdeburg, Magdeburg, Germany; 5 Center for Health and Medical Prevention (CHaMP), Otto-von-Guericke University Magdeburg, Magdeburg, Germany

**Keywords:** *Enterococcus faecalis*, *Enterococcus faecium*, DIA-MS, Proteomics, Bile acids, Resistance mechanisms

## Abstract

**Background:**

All gastrointestinal pathogens, including *Enterococcus faecalis* and *Enterococcus faecium*, undergo adaptation processes during colonization and infection. In this study, we investigated by data-independent acquisition mass spectrometry (DIA-MS) two crucial adaptations of these two *Enterococcus* species at the proteome level. Firstly, we examined the adjustments to cope with bile acid concentrations at 0.05% that the pathogens encounter during a potential gallbladder infection. Therefore, we chose the primary bile acids cholic acid (CA) and chenodeoxycholic acid (CDCA) as well as the secondary bile acid deoxycholic acid (DCA), as these are the most prominent bile acids. Secondly, we investigated the adaptations from an aerobic to a microaerophilic environment, as encountered after oral-fecal infection, in the absence and presence of deoxycholic acid (DCA).

**Results:**

Our findings showed similarities, but also species-specific variations in the response to the different bile acids. Both *Enterococcus* species showed an IC_50_ in the range of 0.01- 0.023% for DCA and CDCA in growth experiments and both species were resistant towards 0.05% CA. DCA and CDCA had a strong effect on down-expression of proteins involved in translation, transcription and replication in *E. faecalis* (424 down-expressed proteins with DCA, 376 down-expressed proteins with CDCA) and in *E. faecium* (362 down-expressed proteins with DCA, 391 down-expressed proteins with CDCA). Proteins commonly significantly altered in their expression in all bile acid treated samples were identified for both species and represent a “general bile acid response”. Among these, various subunits of a V-type ATPase, different ABC-transporters, multi-drug transporters and proteins related to cell wall biogenesis were up-expressed in both species and thus seem to play an essential role in bile acid resistance. Most of the differentially expressed proteins were also identified when *E. faecalis* was incubated with low levels of DCA at microaerophilic conditions instead of aerobic conditions, indicating that adaptations to bile acids and to a microaerophilic atmosphere can occur simultaneously.

**Conclusions:**

Overall, these findings provide a detailed insight into the proteomic stress response of two *Enterococcus* species and help to understand the resistance potential and the stress-coping mechanisms of these important gastrointestinal bacteria.

**Supplementary Information:**

The online version contains supplementary material available at 10.1186/s12866-024-03253-0.

## Introduction

The genus *Enterococcus* is a group of Gram-positive, facultative anaerobic, non-spore-forming, coccal bacteria that were first described in 1899 by MacCallum and Hastings [1, 2]. Usually, various *Enterococcus* species are present in the human gastrointestinal tract, but they are also found in animals and in environmental samples. Some *Enterococcus* species are used as probiotic bacteria or in a variety of dairy products such as cheese or milk [3, 4]. Particularly *Enterococcus faecalis* and *Enterococcus faecium* belong to the natural commensal bacteria of the human intestinal tract [5]. As opportunistic pathogens, they have become a relevant cause for community-acquired and nosocomial infections worldwide [6–8]. Especially *E. faecium* has become one of the most frequently reported sources for life-threatening hospital-acquired infections due to its potential antibiotic resistance to vancomycin and linezolid [9]. As intestinal inhabitants, *E. faecalis* and *E. faecium* are permanently exposed to bile acids. Human bile roughly consists of ∼ 40% cholic acid (CA) and ∼ 40% chenodeoxycholic acid (CDCA), the primary bile acids, as well as ∼ 20% of the secondary bile acids deoxycholic acid (DCA) and, to a minor proportion, lithocholic acid [10, 11]. These bile acids are conjugated with glycine or taurine in the liver cells so that a total of eight possible conjugated bile acids are present [10]. Among the diverse functions of bile is the solubilization and emulsification of fat, which makes bile an important biological detergent [12]. Bile acids specifically alter the glycolipid, phospholipid, and fatty acid composition of bacterial cell membranes [13]. Under exposure to bile acids, bacteria experience problems in maintaining membrane integrity, resulting in increased membrane permeability and leakage of intracellular material. High concentrations of bile acids can rapidly dissolve membrane lipids, leading to the dissociation of integral membrane proteins. This immediate effect leads to leakage of cell contents and cell death. Lower concentrations of bile acids, which are insufficient to directly induce cell death, result in altered activity of critical membrane-bound enzymes and increased transmembrane flux of divalent cations. The rate of bile acid binding to membrane lipids correlates with their hydrophobicity. Conjugated bile acids, being strong acids, are fully ionized at neutral pH and remain in the outer cell membrane. Unconjugated bile acids can passively traverse the lipid bilayer and enter the cell directly. The rate of traversal depends on the number of hydroxyl groups, with dihydroxy bile acids traversing quickly and trihydroxy bile acids traversing more slowly [13]. Moreover, DNA damage may be induced by bile acids [12]. As a consequence, many bacteria that inhabit the gastrointestinal tract have evolved mechanisms to cope with bile acid stress. Genome and transcriptome studies in Gram-positive bacteria have shown, that the expression of genes encoding for transporters that excrete bile acids is regulated by bile acids [14–16]. Other genes that are regulated by the presence of bile are involved in general stress response or carbohydrate metabolism [17].

Enterococci are typical pathogens in cholecystitis and are particularly associated with common bile duct (CBD) stones [18]. They also play a significant role in iatrogenically induced infections such as cholangiopancreatography (ERCP) induced cholangitis [19, 20], acute pancreatitis [21, 22], postoperative pancreatic fistulae [23], and other post-surgery biliary tract infections [24, 25]. In particular, disease progression of primary sclerosing cholangitis (PSC) has been associated with the presence of enterococci [26]. In case of an acute cholecystitis, the bacteria entering the biliary tract must adapt to the high and varying bile acid concentrations up to 17% [27].

Genomic and transcriptomic data have shown interesting results about the bile acid response in *E. faecalis* and *E. faecium.* Transcriptional analyses in *E. faecium* to bile acid stress have identified major changes in the transcriptomic response when analyzed after five and fifteen minutes, where genes involved in nucleotide transport and metabolism were down-regulated [28]. Genes responsible for carbohydrate metabolism and posttranslational modifications, protein turnover and chaperones were found to be up-regulated [29]. Moreover, a study by Solheim et al. in 2007 analyzed the transcriptomic response between 10 and 60 min after bile acid exposure. A high number of genes that are responsible for cell envelope or fatty acid and phospholipid metabolism were repressed, while genes that encode for multidrug-resistance transporters or V-type ATPases were found to be induced [28]. In contrast, only few data on proteomic changes after bile acid exposure exist for *Enterococcus* species. In 2010, Bøhle et al. analyzed the *E. faecalis* proteome with exposure to 1% bovine bile over 20, 60 or 120 min. In mass spectrometric analyses, they found mainly proteins involved in fatty acid and phospholipid biosynthesis pathways to be down-expressed [30]. All of these studies were focused on the effects of bile over a short time period, while studies on the long-term effects are lacking.

Furthermore, data-independent acquisition mass spectrometry (DIA-MS) has not been applied to analyze the *Enterococcus* bile acid response so far, although this technique enables quantitative analysis of every detectable compound in a sample of proteins and thus provides a high reliability in the quantitative results [31]. In this study we used DIA-MS to systematically compare the long-term proteomic changes (18 h) of *E.faecalis* and *E. faecium* after incubation with chenodeoxycholic acid (CDCA) and cholic acid (CA) as primary bile acids, as well as deoxycholic acid (DCA) as a secondary bile acid at concentrations physiologically found in the human biliary tract, assuming a similar stress response in both microbial species.

When considering colonization or infection of the biliary tract by a new fecal-orally transmitted enterococcal strain, the transition from aerobic conditions in the duodenum to microerophilic and finally to anaerobic conditions in the gallbladder must be considered in addition to the bile acid load. Therefore, we conducted a second independent experiment, in which we examined and compared the impact of aerobic and microaerophilic conditions on bile acid stress in *E. faecalis*, both with and without exposure to a low concentration of DCA. This investigation aimed to reveal the potential adaptations of the bacteria to these conditions, highlighting their relevance in scenarios such as fecal-oral uptake of these bacteria, which can occur especially in infants.

## Materials and methods

### *Enterococcus* strains and growth conditions

*E. faecalis* ATCC 700802 (V583) and *Enterococcus faecium* TX0016 (ATCC BAA-472) were grown in M17 broth (Thermo Fisher Scientific, Waltham, Massachusetts, USA), as previous experiments had shown that both organisms exhibit optimal growth in M17 broth [32–34]. Sublethal concentrations of 0.05% CA, CDCA or DCA were added to the medium before incubation. The control sample was grown without bile acids. Stock solutions of 1% sodium-CA, sodium-CDCA and sodium-DCA (Merck, Darmstadt, Germany) were prepared in dH_2_O. Cultures were grown at 37 °C and shaking at 150 rpm, for 18 h, respectively, to obtain samples in the plateau before the stationary phase.

Growth curves were generated by measuring the optical density at 600 nm (OD_600_) every 30 min for the first five hours after inoculation and finally after 24 h. In the growth experiments, biological triplicates of 0%, 0.01%, 0.025%, 0.038% and 0.05% of either DCA, CA or CDCA were analyzed. The IC_50_ was determined with GraphPad Prism version 6 (GraphPad Software, La Jolla, California, USA) using nonlinear regression with the model Y = Bottom + (Top - Bottom)/(1 + 10^((LogIC_50_-X)*HillSlope)). Top represents the maximum response, Bottom is the minimal response.

To analyze the adaptation to microaerophilic conditions, bacteria were first grown in normal atmosphere and then diluted to an OD_600_ of 0.05 and incubated in parallel for 18 h either in normal atmosphere or under microaerophilic conditions with and without 0.01% DCA, respectively. The OD_600_ was measured every hour for six hours and after 24 h. The microaerophilic environment was created using the BBL CampyPak Plus microaerophilic system (Becton Dickinson, Franklin Lakes, New Jersey, USA).

### Protein purification and quantification

After 18 h of growth in liquid M17 medium at 37 °C and shaking at 150 rpm, the cultures were transferred to ice immediately and protein purification was started. Cultures were centrifuged at 3,500 x*g* for 10 min at 4 °C. Afterwards, the cells were resuspended in 1 mL 0.9% NaCl aqueous solution, which is commonly used as a buffer due to its osmotic balance function, to preserve macromolecules such as proteins. In the next step, 0.75 g 4 mm glass beads were added, and samples were treated in a “Fast prep 96 Homogenizer” (MP Biomedicals Germany GmbH, Eschwege, Germany) for 2 × 20 s, followed by centrifugation at 5,500 x*g* for one minute. The supernatant was removed, and the samples were centrifuged at 13,500 x*g* for 10 min at 4 °C. The supernatant was taken and used for further procedures.

A Pierce assay (Thermo Fisher Scientific, Waltham, Massachusetts, USA) was used to determine the protein concentration in each sample. For DIA-MS analysis, concentrations were adjusted to 1 µg/µL of protein. All samples were prepared in triplicate.

### DIA-MS

DIA-MS analysis was selected to identify proteins from the samples because of its exceptional reliability and reproducibility, allowing for the acquisition of meaningful proteomic profiles. In comparison to other methods like 2D-Gel analysis, DIA-MS offers a more comprehensive and consistent analysis, particularly in terms of the number of proteins that can be identified. In addition, our own preliminary experiments with SILAC (stable isotope labeling by/with amino acids) have shown that the stable isotopically labeled amino acids are only poorly incorporated into bacteria that do not exhibit auxotrophy for the corresponding amino acid. Therefore these methods exhibiting a lower sensitivity, regarding identification and quantification of proteins. Samples were purified by short-run SDS-PAGE with Coomassie stain (in-gel tryptic digestion). For the library, a pre-fractionation of a pooled reference sample was divided into 12 fractions by basic pH-reversed phase chromatography. Spiking was performed with a Biognosys iRT peptide standard.

For mass spectrometric analysis, identification was done by data-dependent acquisition (DDA) on a TripleTOF 5600+ (Sciex, Darmstadt, Germany). Therefore, 1 mg equivalent were loaded, followed by a 90 min gradient, and the Top25 method. Two technical replicates were made per RP fraction. Quantification and ID by DIA-MS were performed using Thermo Q Exactive. Three technical replicates per sample were prepared. Data processing was done with the Spectronaut v16.0.220606.53000 software package (Biognosys AG, Schlieren, Switzerland).

Protein identification and hybrid spectral library generation from 12 × 2 DDA acquisitions and 12 × 2 DIA acquisitions experiments were performed using Pulsar search engine against UniProtKB *E. faecalis* 700802 and *E. faecium* TX0016 proteomes with default parameters. A False Discovery Rate (FDR) of 1% on the spectral, peptide and protein group levels was set for all samples. DIA quantification was done using up to 6 fragments per peptide and up to 10 peptides per proteins. Dynamic retention time alignment was done, as well as dynamic mass recalibration and quartile normalization, for 1% FDR. Global data imputation was done for the final results table.

### Data processing

The mass spectrometry proteomics data have been deposited to the ProteomeXchange Consortium via the PRIDE [35–37] partner repository with the dataset identifier PXD040819. For statistical analysis, Perseus v1.6.2.2 was used to generate volcano plots for comparison between different samples [38]. Two-fold expression changes were defined as significant. Only proteins that were regulated in five out of six samples were considered. For generation of volcano-plots in Perseus, a t-test was chosen with a number of randomizations = 250 and a FDR = 0.05 [39]. If not otherwise stated, all proteins that are subsequently described as up- or down-expressed were significantly regulated.

The respective COG-categories were assigned to the proteins using eggNOGmapper v 2.18 [40–42]. Venn diagrams were generated using InteractiVenn [43] to identify proteins that were consistently up- or down regulated in all bile acid treated samples. For comparison, the whole theoretical proteome from UniProtKB was used for both organisms. Growth-curves, donut-plots and heatmaps were generated using matplotlib in python3 [71].

## Results

### Growth phenotype comparison between *E. faecalis* and *E. faecium* in the presence of DCA, CDCA and CA

**Fig. 1:**
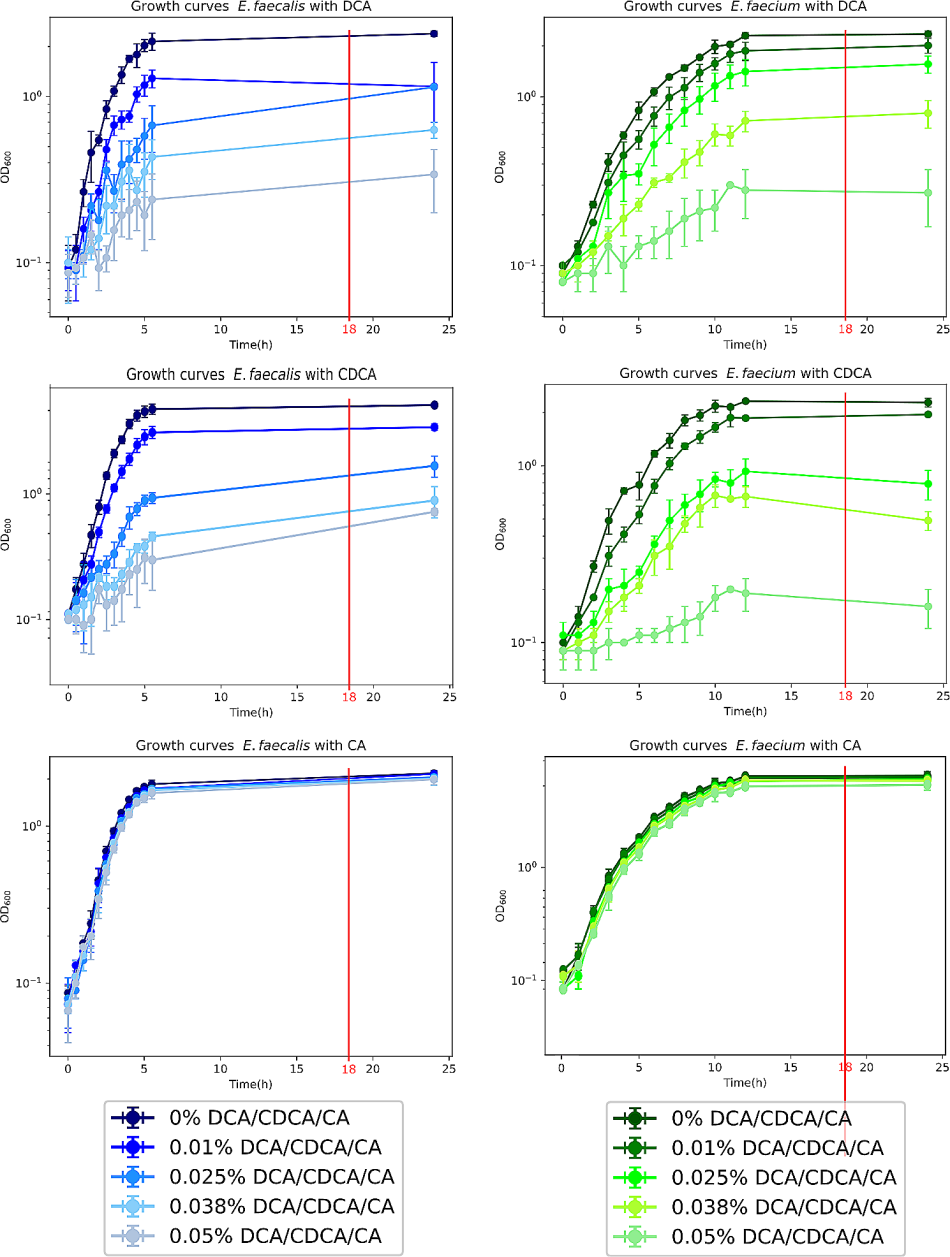
Growth curves of *E. faecalis* (blue) and *E. faecium* (green) with DCA, CDCA and CA at 0%, 0.01%, 0.025%, 0.038% and 0.05% bile acid concentration. The OD_600_ was measured every half hour for 5.5 h and after 24 h for *E. faecalis* and every hour for 12 h and after 24 h for *E. faecium*. The OD_600_ on the Y-axis is shown in a logarithmic scale

We compared the growth phenotypes of *E. faecalis* and *E. faecium* in the presence of 0%, 0.01%, 0.025%, 0.038% and 0.05% of DCA, CDCA and CA, respectively. Growth gradually decreased with increasing DCA and CDCA concentrations (Fig. 1). At 0.05% DCA and CDCA, only a weak increase of the OD_600_ was detectable after 24 h (Fig. 1), indicating a strong inhibitory effect. The IC_50_ for DCA and CDCA was similar for both species and in the range of 0.01–0.023% when determined at three different time points at 3 h, 5.5 h and 24 h (Table 1). In contrast, growth curves were almost unaffected by CA in both species, even at the highest concentration of 0.05% (Fig. 1), suggesting a high resistance of both *Enterococcus* species towards this primary bile acid.

**Table 1:** IC_50_ of *E. faecalis* and *E. faecium* after 3, 5.5 and 24 h of growth in the three different bile acids. IC_50_ was determined via graph pad prism after measurement of the OD_600_

		IC_50_*E. faecalis*	IC_50_*E. faecium*
after 3 h of growth	DCA	0.01%	0.015%
	CDCA	0.011%	0.013%
after 5.5 h of growth	DCA	0.012%	0.011%
	CDCA	0.013%	0.014%
after 24 h of growth	DCA	0.011%	0.013%
	CDCA	0.014%	0.023%

### Proteomic stress response towards DCA, CDCA and CA in *E. faecalis* and *E. faecium*

The similar sensitivity pattern of *E. faecalis* and *E. faecium* towards the three tested bile acids leads to the assumption that their adaptation processes are likely to be similar, as well. To investigate the involved stress response more thoroughly, we decided to analyze the proteome profile changes of *E. faecalis* and *E. faecium* after individual exposure with the three bile acids (0.05% for 24 h) in comparison to an untreated control. The concentration of bile acids was deliberately chosen to simulate a proteome under significant stress, similar to the concentrations encountered in the gallbladder environment during colonization of this organ, where bile concentrations can vary, depending on different factors such as diet and diseases [27, 44]. For *E. faecalis* samples, a total of 1,410 proteins were identified in DIA-MS which represented 43.5% of the whole theoretical proteome. 1,400 proteins were identified for *E. faecium* samples, which represented 45.8% of the whole theoretical proteome (Table 2).

**Table 2:** Number of up- or down-expressed proteins of *E. faecalis* and *E. faecium* in three different bile acids and the respective percentage amount of the total identified proteins in DIA-MS

	DCA		CDCA		CA	
	Up-expressed	down-expressed	up-expressed	down-expressed	up-expressed	down-expressed
*E. faecalis*	207/1410 (15%)	424/1410 (30%)	232/1410 (16%)	376/1410 (27%)	264/1410 (19%)	380/1410 (27%)
	631/1410 (45%)		608/1410 (43%)		644/1410 (46%)	
*E. faecium*	260/1400 (19%)	362/1400 (26%)	174/1400 (12%)	391/1400 (28%)	409/1400 (29%)	224/1400 (16%)
	622/1400 (44%)		565/1400 (40%)		633/1400 (45%)	

The number of proteins with significantly altered expression level was similar in all bile acid treated samples in both organisms. DCA resulted in 631 differentially expressed proteins in *E. faecalis* and 622 in *E. faecium*. CDCA treatment resulted in 608 differentially expressed proteins in *E. faecalis* and 565 in *E. faecium*. Interestingly, after CA exposure the number of differentially expressed *E. faecalis* proteins (644) and *E. faecium* proteins (633) was in the same range as with DCA and CDCA, although the latter bile acids mediated a markedly stronger growth inhibition (Table 2; Fig. 1).

When differentially expressed proteins were separated into up-expressed and down-expressed proteins, the number of down-expressed proteins exceeded the number of up-expressed proteins (Table 2). For example, the fraction of down-expressed proteins on the overall differentially expressed proteins was 67% for DCA, 62% for CDCA and 58% for CA in *E. faecalis*.

### Clusters of orthologous groups of proteins (COG) categories

**Fig. 2:**
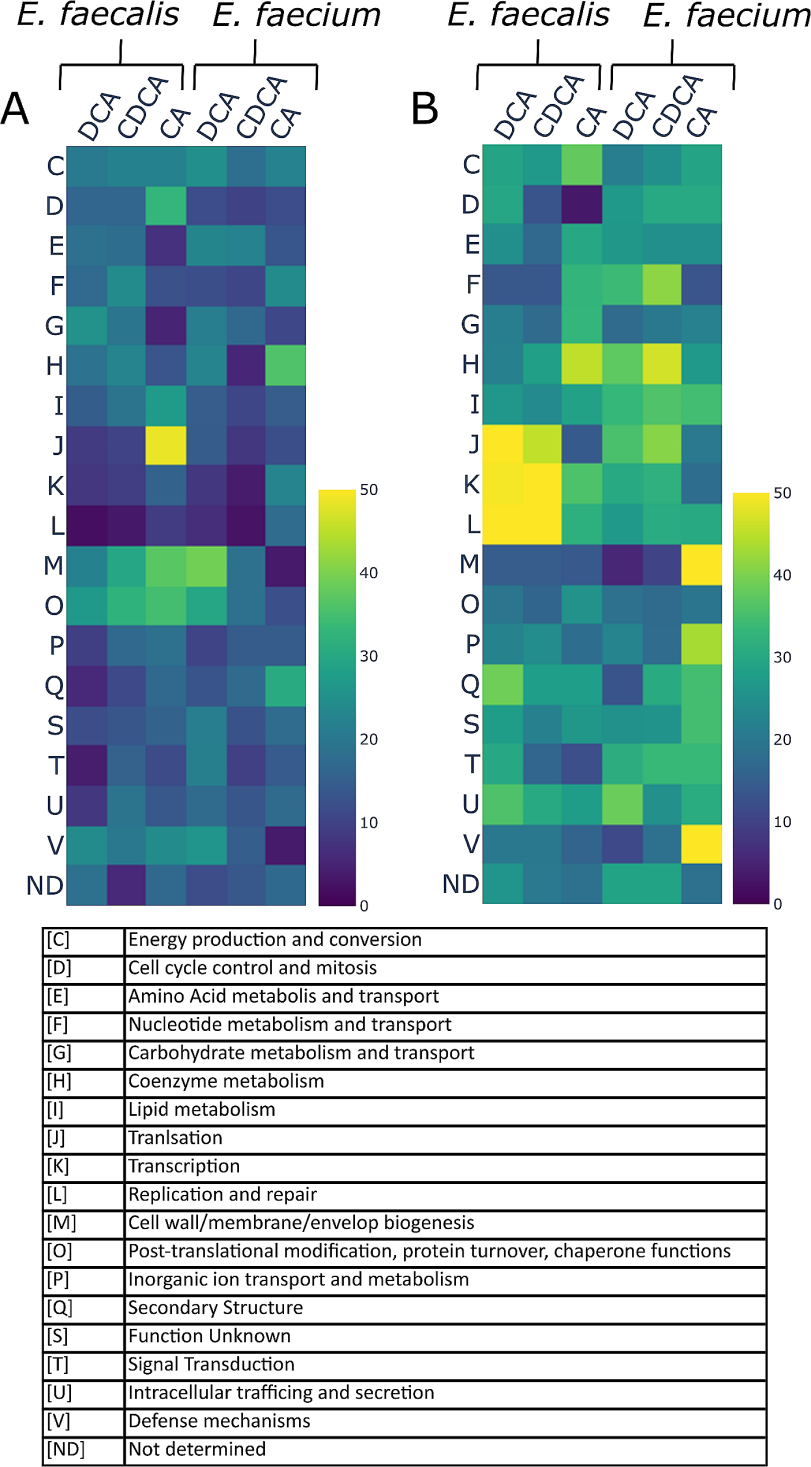
The total number of detected proteins as well as the regulated proteins were assigned to their respective COG-category. The percentage of regulated proteins in relation to the total number was calculated for each COG-category and visualized in a heatmap. A: Up-expressed proteins B: down-expressed proteins. Yellow = 40–50%, green = 25–40%, bright blue = 10–25%, darkblue = 0–10% higher than in the whole proteome

Differentially expressed proteins were assigned to their respective COG categories and significantly up- or down-expressed proteins were depicted in doughnut plots (Suppl. Figure 1). The relative proportion of the individual COG-categories showed a species-specific pattern. We furthermore determined the proportion of up- and down-expressed proteins for each bile acid within the individual COG-categories (Fig. 2).

**Fig. 3:**
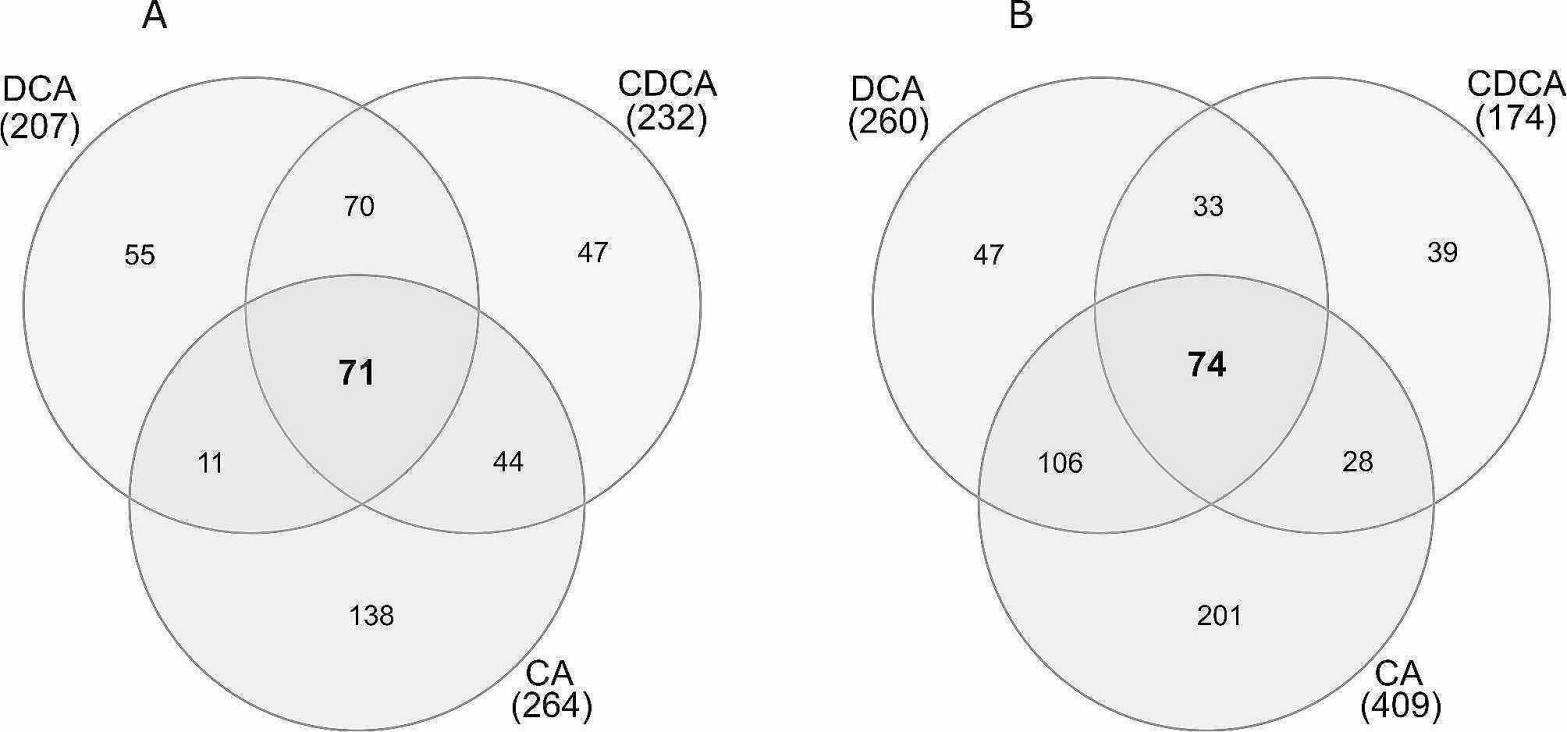
Venn diagrams of proteins that are commonly up-expressed in all approaches with 0.05% bile salts in *E. faecalis* (A) and *E. faecium* (B). In *E. faecalis*, 71 proteins are commonly up-expressed, while in *E. faecium*, 74 proteins are commonly upexpressed

**Fig. 4:**
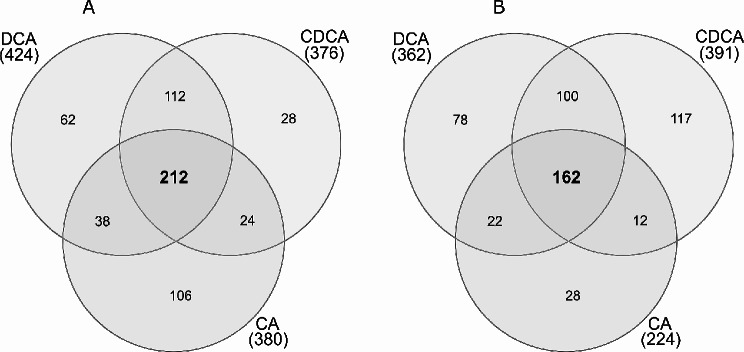
Venn diagrams of proteins that are commonly down-expressed in all approaches with 0.05% bile salts in *E. faecalis* (A) and *E. faecium* (B). In *E. faecalis*, 212 proteins are parallel down-expressed, while in *E. faecium*, 162 proteins are commonly down-expressed

Independent from the *Enterococcu*s species the response to DCA showed higher similarity to the CDCA response than to the response towards the more hydrophilic primary bile acid CA. The number of commonly up-expressed proteins by all three bile acids was similar for *E. faecalis* (71) and *E. faecium* (74). Likewise, the number of commonly down-expressed proteins was 212 for *E. faecalis* and 162 for *E. faecium* (Figs. 3 and 4). The distribution of these proteins in COG categories was different, suggesting that the general bile stress response varied between the two microbial species (Suppl. Figure 1). In *E. faecalis*, DCA and CDCA resulted in down-expression of around 50% of proteins in the COG categories “translation” (J), “transcription” (K) and “replication” (L), as shown in Fig. 2. These three categories were also down-expressed in *E. faecium*, but to a lesser extent, between 30% and 40% of the proteins assigned to these categories were down-expressed (Fig. 2). This suggested that *E. faecium* was more tolerant towards DCA/CDCA stress than *E. faecalis*.

In contrast, CA showed a less pronounced effect on the COG categories “translation (J), “transcription” (K) and “replication” (L), in *E. faecalis* and *E. faecium*. Only 10–20% of the proteins of the respective categories were down-expressed in both organisms. This was in accordance with the different growth phenotype in the presence of CA when compared to DCA or CDCA in both organisms. The proteins of the categories ”cell wall/membrane/envelope biogenesis” (M) and “post-translational modification, protein turnover, and chaperones” (O) were relatively high up-expressed under bile acid stress conditions. Between 30% and 40% of the identified proteins belonging to these categories were up-expressed (Fig. 2) in both microbial species.

### *E. faecalis* in microaerophilic vs. aerobic conditions, with and without DCA exposure

**Fig. 5:**
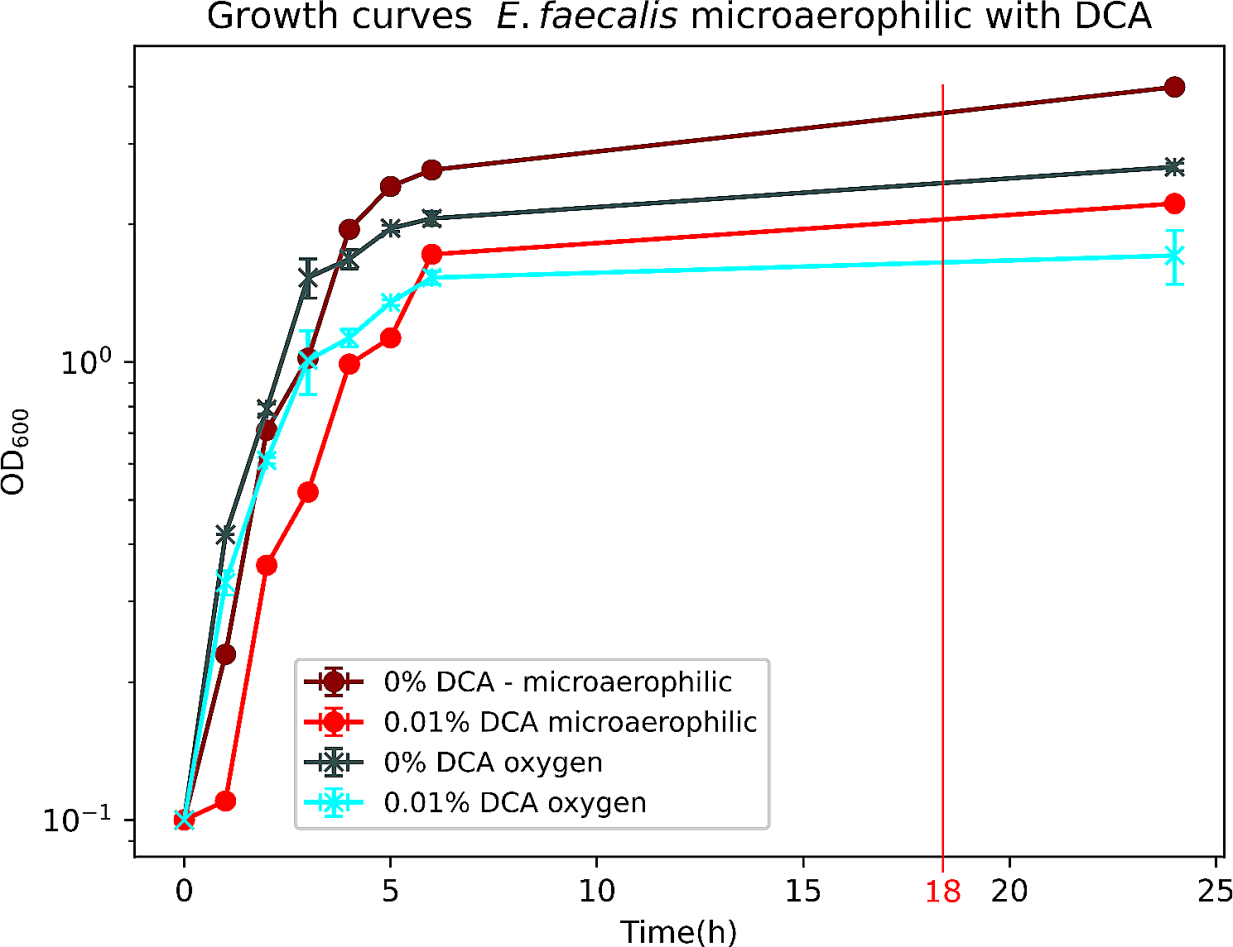
Growth curves of *E. faecalis* with (blue) and without (red) oxygen and with (bright colors) and without 0.01% DCA (dark colors). The OD_600_ was measured every hour for six hours and after 24 h. After 24 h, growth was higher in microaerophilic conditions than with normal oxygen concentration

As an intestinal inhabitant, *E. faecalis* is adapted to microaerophilic and anaerobic habitats. However, in case of an oral uptake of *E. faecalis*, potentially originating from fecal sources, the bacteria must undergo adaptations to transition from aerobic to microaerophilic and anaerobic conditions. Moreover, the bacteria are exposed to bile acid in presence and absence of oxygen in the different environments of the gastrointestinal tract. We thus compared the *E. faecalis* growth phenotype and its alterations of the proteome in aerobic versus microaerophilic conditions in an independent experiment. *E. faecalis* displayed a similar growth dynamics under both atmospheric conditions up to 6 h. However, at 24 h a markedly higher final OD_600_ of ≈ 4.0/2.2 was measured under microaerophilic conditions than with normal oxygen concentration (OD_600_ ≈ 2.7/1.7, Fig. 5).

**Fig. 6:**
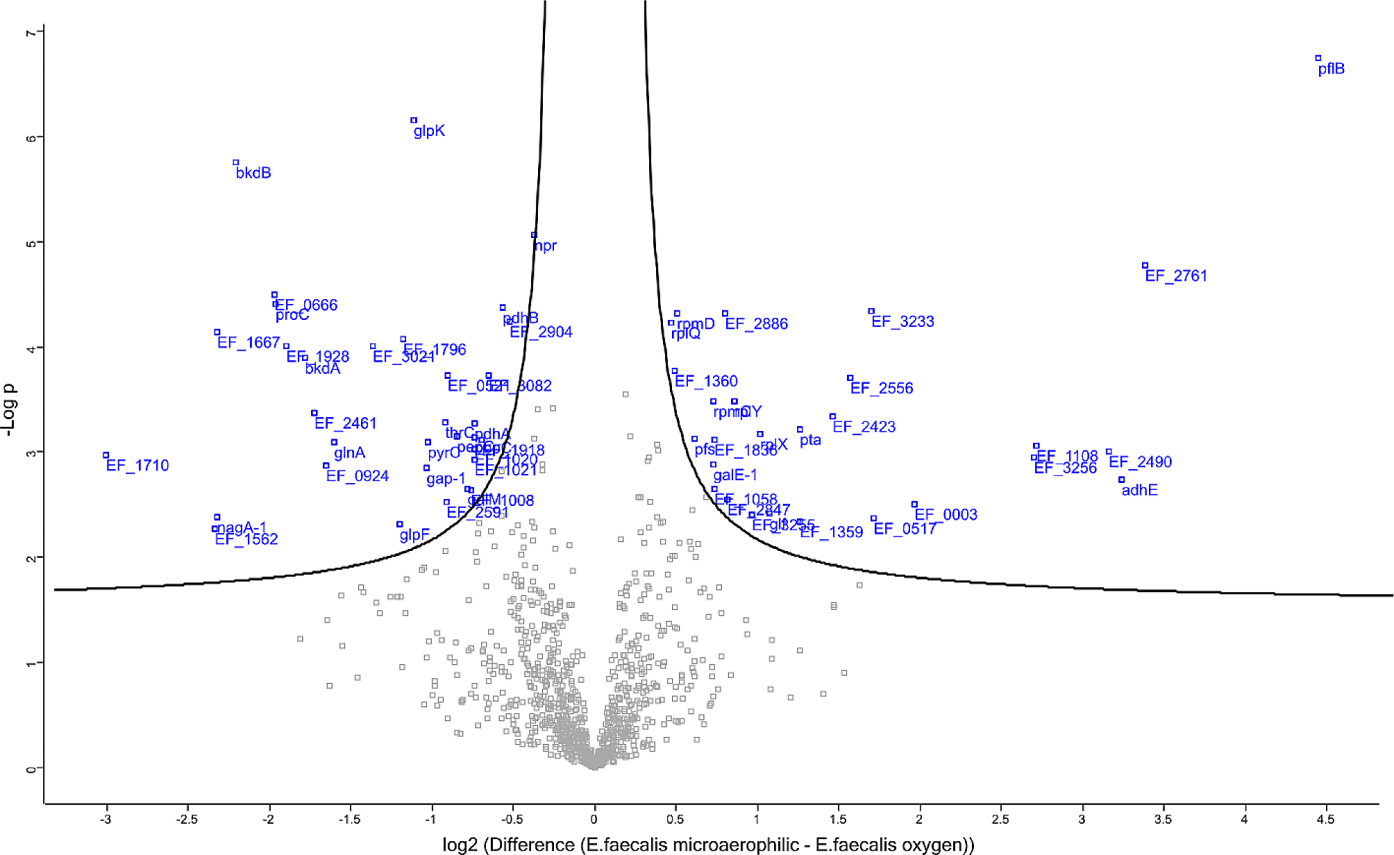
Volcano-plot of *E. faecalis* grown in normal oxygen concentration compared to microaerophilic conditions. The X-axis shows the scale of difference between both proteomes and the Y-axis shows the -log of the p-value. Blue marked squares represent significantly up-expressed proteins in the different conditions. On the left side of the volcano, the proteins of the up-expressed proteins of samples grown in microaerophilic conditions are shown and on the right side, up-expressed proteins of the samples grown in aerobic conditions are shown

Proteomic analysis revealed 59 differentially expressed proteins in response to a microaerophilic atmosphere, with 27 up-expressed and 32 down-expressed proteins under microaerophilic conditions compared to aerobic conditions (Tables 3 and 4; Fig. 6). In samples grown under microaerophilic conditions, several ribosomal proteins were up-expressed compared to normal oxygen concentration. On the other hand, various proteins involved in glycolysis and carbohydrate catabolism were down-expressed under microaerophilic conditions, for example glyceraldehyde-3-phosphate dehydrogenase, components of the pyruvate dehydrogenase complex, an aldose epimerase, and a glycosyl hydrolase family protein.

**Table 3:** Up-expressed proteins in *E. faecalis* under microaerophilic conditions when compared to aerobic conditions with their respective difference (fold-change values). Bold-marked proteins are constituents of ribosomes

Protein names	Difference value	Potential function
Q834N1_ENTFA	4.4478E + 14	Formate acetyltransferase
Q830L9_ENTFA	3.382E + 14	PSP1 C-terminal domain-containing protein
Q837E3_ENTFA	3.2351E + 14	Aldehyde-alcohol dehydrogenase
Q831L4_ENTFA	3.1573E + 14	Uncharacterized protein
Q82Z23_ENTFA	2.699E + 14	Pheromone cAD1 lipoprotein
Q839Z3_ENTFA	1.9673E + 14	S4 RNA-binding domain-containing protein
Q82Z45_ENTFA	1.6975E + 14	Dps family protein
Q831F4_ENTFA	1.5673E + 14	Fumarate reductase flavoprotein subunit. putative
Q831S7_ENTFA	1.4604E + 14	Transcriptional regulator. ArsR family
Q835L8_ENTFA	1.263E + 14	Phosphoenolpyruvate–glycerone phosphotransferase
Q836Z4_ENTFA	1.2599E + 14	Phosphotransacetylase
Q831L7_ENTFA	1.0706E + 14	UDP-galactopyranose mutase
**RL24_ENTFA**	**1.0172E + 14**	**50 S ribosomal protein L24**
Q836K3_ENTFA	2.7148E + 13	Oxidoreductase. putative
H7C6Z5_ENTFA	1.7103E + 13	2-dehydropantoate 2-reductase
Q82Z24_ENTFA	0.96505372	FAD:protein FMN transferase
**RL25_ENTFA**	**0.85790229**	**50 S ribosomal protein L25**
Q830E0_ENTFA	0.8144203	Uncharacterized protein
Q830A9_ENTFA	0.79726458	Transcriptional regulator. MarR family
Q836Q0_ENTFA	0.7379541	Universal stress protein family
Q833U2_ENTFA	0.7335097	PTS system. IIA component. putative
Q836N9_ENTFA	0.72549907	UDP-glucose 4-epimerase
**RL29_ENTFA**	**0.72478835**	**50 S ribosomal protein L29**
Q830S8_ENTFA	0.61513432	5’-methylthioadenosine/S-adenosylhomocysteine nucleosidase
**RL30_ENTFA**	**0.50778174**	**50 S ribosomal protein L30**
Q835L7_ENTFA	0.48959955	Dihydroxyacetone kinase family protein
**RL17_ENTFA**	**0.46598546**	**50 S ribosomal protein L17**

**Table 4:** Down-expressed proteins of *E. faecalis* in microaerophilic conditions when compared to aerobic conditions with their respective difference (fold-change values). Bold-marked proteins represent proteins involved in glycolysis and pyruvate metabolism

Protein names	Difference value	Potential function
Q834E5_ENTFA	-3.007E + 14	Transcriptional regulator. LysR family
H7C796_ENTFA	-2.339E + 14	Phospho-2-dehydro-3-deoxyheptonate aldolase. putative
Q834I5_ENTFA	-2.322E + 14	Short chain dehydrogenase family protein
**Q835Q8_ENTFA**	**-2.319E + 14**	*N*-**acetylglucosamine-6-phosphate deacetylase**
H7C710_ENTFA	-2.204E + 14	Branched-chain alpha-keto acid dehydrogenase. E1 component. beta subunit
Q838A6_ENTFA	-1.971E + 14	Glyoxalase family protein
Q831S6_ENTFA	-1.963E + 14	Pyrroline-5-carboxylate reductase
Q833L7_ENTFA	-1.897E + 14	Alpha-glycerophosphate oxidase
**Q834J1_ENTFA**	**-1.782E + 14**	**Branched-chain alpha-keto acid dehydrogenase. E1 component. alpha subunit**
Q831P0_ENTFA	-1.729E + 14	Inositol monophosphatase protein family
Q837B9_ENTFA	-1.65E + 14	Uncharacterized protein
Q832R0_ENTFA	-1.603E + 14	Glutamine synthetase
Q82ZN0_ENTFA	-1.365E + 14	Uncharacterized protein
Q833L8_ENTFA	-1.198E + 14	Glycerol uptake facilitator protein
Q833 × 8_ENTFA	-1.176E + 14	Lipoprotein. putative
**GLPK_ENTFA**	**-1.114E + 14**	**Glycerol kinase**
**Q834V5_ENTFA**	**-1.037E + 14**	**Glyceraldehyde-3-phosphate dehydrogenase**
PYRC_ENTFA	-1.03E + 14	Dihydroorotase
Q831S9_ENTFA	-0.9186293	Threonine synthase
Q831C0_ENTFA	-0.9158467	Glyoxalase family protein
H7C725_ENTFA	-0.9020282	Choloylglycine hydrolase family protein
Q838Y1_ENTFA	-0.8457337	Aminopeptidase
**Q836P1_ENTFA**	**-0.7834327**	**Aldose 1-epimerase**
Q836U8_ENTFA	-0.757721	Oxidoreductase. Gfo/Idh/MocA family
Q836V7_ENTFA	-0.741525	Penicillin-binding protein C
Q836T7_ENTFA	-0.7396049	Glycosyl hydrolase. family 1
**Q835M4_ENTFA**	**-0.7384612**	**Pyruvate dehydrogenase E1 component subunit alpha**
**Q836T6_ENTFA**	**-0.7364024**	*N*-**acetyltransferase domain-containing protein**
Q833M6_ENTFA	-0.6937323	Uncharacterized protein
Q82ZH5_ENTFA	-0.6532336	Iron compound ABC transporter. substrate-binding protein
**Q835M3_ENTFA**	**-0.5647918**	**Pyruvate dehydrogenase complex. E1 component. beta subunit**
Q82ZZ3_ENTFA	-0.5268988	Lactamase_B domain-containing protein

Treatment with 0.01% DCA resulted in a moderate growth inhibition compared to untreated controls, in both, microaerophilic and aerobic conditions (Figs. 1 and 5). As observed for DCA-untreated samples, DCA-treated samples also displayed increased growth under microaerophilic conditions compared to aerobic conditions after 24 h (Fig. 5). Proteome analysis revealed that under aerobic conditions, 419 proteins were up-expressed and 245 down-expressed at 0.01% DCA, compared to DCA-untreated controls grown at aerobic conditions. Similarly, in microaerophilic conditions, 396 proteins were up-expressed, and 251 proteins were down-expressed in 0.01% DCA treated samples compared to DCA-untreated controls grown at microaerophilic conditions (Table 5). Of the up-expressed proteins, 90 proteins were also identified in the 0.05% DCA samples, among these were for example several V-type ATPase subunits, ABC-transporters and murein-synthesis related proteins. All in all, the differential expression of proteins at 0.01% of DCA was highly similar to the protein expression at 0.05% DCA. This similarity implies that the impact on protein expression is consistent across these two concentrations of DCA and suggests that the biological processes may be particularly responsive to the presence of DCA.

**Table 5:** The number of up- or down-expressed proteins in aerobe and microaerophilic *E. faecalis* approaches with 0.01% DCA and the respective percentage amount of the total identified proteins in DIA-MS

	Aerobic + DCA		Microaerophilic + DCA	
	up	down	up	down
*E. faecalis*	419/1050 (40%)	245/1050 (23%)	396/1050 (38%)	251/1050 (24%)
	664/1050 (63%)		647/1050 (62%)	

Interestingly, the 0.01% DCA-treated samples under microaerophilic conditions showed 46 of the 59 differentially expressed proteins that were identified in the DCA untreated sample under microaerophilic conditions (Suppl. Figure 2). This indicates that DCA stress does not prevent the up- and down-expression of the majority of proteins that occur as an adaptation to microaerophilic conditions.

### Identification of a general bile stress response based on *E. faecalis* and *E. faecium* proteins commonly significantly altered in their expression

As described above, treatment with 0.05% DCA, CDCA, or CA identified 71 commonly up-expressed proteins. Proteomic data for the approaches with 0.05% and with 0.01% DCA were obtained from independent experiments performed at different time points and can thus not be directly compared. Nevertheless, of the 71 commonly up-expressed proteins identified from the 0.05% bile acid samples, 37 proteins were also up-expressed in the two samples using 0.01% DCA with either aerobic or microaerophilic atmosphere (Tables 6 and 7, Suppl. Figure 3). This suggests a strong conservation of the general stress response towards DCA, independent from atmospheric conditions.

**Table 6:** 37 proteins which were commonly up-expressed in all five *E. faecalis* approaches in the presence of 0.05% DCA, CDCA or CA and 0.01% DCA under aerobic as well as microaerophilic conditions. Proteins involved in murein or peptidoglycan synthesis are marked in Italic, transporter proteins are marked in Bold and V-type ATPase subunits are marked in bolditalics

‍Uniprot ID	Protein function
*H7C6V7_ENTFA*	*Penicillin-binding protein 4*
H7C713_ENTFA	Cell division protein DivIVA
Q82YZ9_ENTFA	Peptidase, U32 family, putative
Q82ZA8_ENTFA	Hydrolase, haloacid dehalogenase-like family
**Q82ZH5_ENTFA**	**Iron compound ABC transporter, substrate-binding protein**
Y2866_ENTFA	Probable transcriptional regulatory protein EF_2866
Q830N7_ENTFA	Lipoate–protein ligase
Q830 × 4_ENTFA	Diacylglycerol kinase catalytic domain protein
**Q831B8_ENTFA**	**ABC transporter, ATP-binding/permease protein**
**Q831B9_ENTFA**	**ABC transporter, ATP-binding/permease protein**
RF1_ENTFA	Peptide chain release factor 1
Q831R2_ENTFA	PTS system, IIA component
EFTS_ENTFA	Elongation factor Ts
Q832A0_ENTFA	Uncharacterized protein
Q832N1_ENTFA	dTDP-glucose 4,6-dehydratase
Q833B2_ENTFA	Oxidoreductase, pyridine nucleotide-disulfide family
**MURC_ENTFA**	UDP-*N*-acetylmuramate–L-alanine ligase
Q834B6_ENTFA	DUF4097 domain-containing protein
Q834G9_ENTFA	DegV family protein, putative
Q834T0_ENTFA	TPR domain protein
***VATB_ENTFA***	***V-type ATP synthase beta chain***
***VATA_ENTFA***	***V-type ATP synthase alpha chain***
***Q834Y2_ENTFA***	***V-type ATPase, subunit E***
***Q834Y4_ENTFA***	***V-type ATP synthase subunit I***
DNAK_ENTFA	Chaperone protein DnaK
GRPE_ENTFA	Protein GrpE
Q835V8_ENTFA	Sulfatase domain protein
*MURA1_ENTFA*	*UDP*-*N*-*acetylglucosamine 1-carboxyvinyltransferase 1*
QUEA_ENTFA	S-adenosylmethionine:tRNA ribosyltransferase-isomerase
*Q837J3_ENTFA*	*UDP*-*N*-*acetylmuramoyl-tripeptide–D-alanyl-D-alanine ligase*
TIG_ENTFA	Trigger factor
Q838M3_ENTFA	Transcriptional regulator, MerR family
Q838M4_ENTFA	Drug resistance transporter, EmrB/QacA family protein
Q838M5_ENTFA	Uncharacterized protein
Q838Q5_ENTFA	Abhydrolase_3 domain-containing protein
EFP_ENTFA	Elongation factor P
EFTU_ENTFA	Elongation factor Tu

**Table 7:** 24 proteins which were commonly down-expressed in all five *E. faecalis* approaches in the presence of 0.05% DCA, CDCA or CA and 0.01% DCA under aerobic as well as microaerophilic conditions. Proteins associated with pyruvate and citrate metabolism are marked in italics, proteins involved in biosynthesis of folic acid and amino acids are marked in bold

‍Uniprot ID	Protein function
**H7C718_ENTFA**	**Single-stranded DNA-binding protein**
*AROA_ENTFA*	*3-phosphoshikimate 1-carboxyvinyltransferase*
*Q82YW0_ENTFA*	*Citrate [pro-3 S]-lyase] ligase*
*Q82Z79_ENTFA*	*Isochorismatase family protein*
Q82ZD3_ENTFA	Uncharacterized protein
Q82ZF0_ENTFA	Peptide ABC transporter, ATP-binding protein
Q82ZF1_ENTFA	Peptide ABC transporter, ATP-binding protein
Q82ZF2_ENTFA	Peptide ABC transporter, permease protein
Q82ZK6_ENTFA	Phosphosugar-binding transcriptional regulator, RpiR family, putative
Q830J7_ENTFA	NAD_binding_9 domain-containing protein
Q831L7_ENTFA	UDP-galactopyranose mutase
Q833L4_ENTFA	Uncharacterized protein
*Q834I9_ENTFA*	*Branched-chain phosphotransacylase*
*Q834J0_ENTFA*	*Dihydrolipoyl dehydrogenase*
**Q834J2_ENTFA**	**Dihydrolipoamide acetyltransferase component of pyruvate dehydrogenase complex**
**Q834R2_ENTFA**	**Dihydrofolate reductase**
Q834W2_ENTFA	PTS system, IIABC components
**Q835H7_ENTFA**	**Cadmium-translocating P-type ATPase**
**DAPA_ENTFA**	**4-hydroxy-tetrahydrodipicolinate synthase**
Q836S2_ENTFA	Nucleoside diphosphate kinase
Q836T6_ENTFA	*N*-acetyltransferase domain-containing protein
Q837A3_ENTFA	Uncharacterized protein
Q837H3_ENTFA	Glyoxalase family protein

Of the 37 up-expressed proteins, four proteins were subunits of a V-type ATP synthase (Table 8), namely alpha chain, beta chain, subunit E and subunit I, suggesting an important role of this protein complex in bile acid stress adaptation (Table 6). In total, nine *E. faecalis* V-type ATPase related proteins were identified in samples with 0.05% of DCA, CDCA or CA. In *E. faecium*, eight V-type ATPase associated proteins were detected in total. However, these proteins were not as frequently up-expressed during bile acid stress as in *E. faecalis*, and only one (V-type ATPase subunit F) was up-expressed in all three bile acids (Table 8, Suppl. Excel file 1). Functional analysis of V-type ATPases in bile acid stress adaptation would greatly benefit from the availability of specific inhibitors for this protein class. In contrast to eukaryotes, specific V-type ATPase inhibitors were not described for prokaryotes yet. In eukaryotic cells, bafilomycin A and archazolid A were shown to act as V-type ATPase inhibitors [45–47]. We tested these compounds in growth assays up to a concentration of 10 µM on *E. faecalis* but could not find any inhibitory effect (data not shown). Furthermore, a combination of 10 µM bafilomycin or archazolid with 0.01% DCA did not lead to stronger growth inhibition as the 0.01% DCA control, indicating that these compounds do not inhibit the bile acid adaptation in *E. faecalis*.

**Table 8:** V-type ATPases identified in *E. faecalis* and *E. faecium* samples with 0.05% bile acids and *E. faecalis* samples with 0.01% DCA in aerobic and microaerophilic conditions. Up-expressed proteins are labelled in grey. Proteins that were not regulated are labeled in white. n.i. = not identified in DIA-MS. ^1^ = absent in genome

	*E. faecalis*			*E. faecium*			*E. faecalis*	
Identified protein	0.05% DCA	0.05% CDCA	0.05% CA	0.05% DCA	0.05% CDCA	0.05% CA	0.01% DCA (aerobe)	0.01% (microaerophilic)
V-type ATP synthase alpha chain	+	+	+	-	+	+	+	+
V-type ATP synthase beta chain	+	+	+	-	+	+	+	+
V-type ATPase subunit C	-	+	+	+	-	+	+	+
V-type ATP synthase subunit D	-	+	+	-	-	-	+	+
V-type ATPase subunit E	+	+	+	-	+	+	+	+
V-type ATPase subunit F	-	-	-	+	+	+	+	+
V-type ATPase subunit G	-	-	+	absent ^1^	absent ^1^	absent ^1^	n.i.	n.i.
V-type ATP synthase subunit I	+	+	+	+	-	+	+	+
V-type ATPase subunit K	-	-	-	-	+	+	+	+

A unique pattern seen in both species was the up-expression of membrane transporters. In *E. faecalis*, three ABC-transporters and one multidrug-resistance transporter were commonly up-expressed in all bile acid treated samples (Table 6). In *E. faecium*, five ABC-transporter and one multidrug-resistance systems were collectively up-expressed (Suppl. Excel file 1).

Furthermore, four proteins involved in peptidoglycan metabolism and murein synthesis were up-expressed in *E. faecalis*. These are a UDP-*N*-acetylmuramate–L-alanine ligase, a UDP-*N*-acetylglucosamine 1-carboxyvinyltransferase 1, a UDP-N-acetylmuramoyl-tripeptide–D-alanyl-D-alanine ligase and a penicillin-binding protein (Table 6). These proteins were also up-expressed in *E. faecium* after exposure with DCA, CDCA, or CA (Suppl. Excel file 1).

Among the 24 down-expressed proteins were central elements of the pyruvate and citrate metabolism, including two components of the pyruvate dehydrogenase complex (dihydrolipoyl dehydrogenase; dihydrolipoamide acetyltransferase) and a [citrate [pro-3 S]-lyase] ligase, which is involved in the cleavage of citrate into acetate and oxaloacetate. Furthermore, down-expression of a key enzyme of the shikimate pathway (AROA_ENTFA) indicates decreased biosynthesis of folates and amino acids. This is in line with reduced expression levels of dihydrofolate reductase, also involved in folate metabolism and of 4-hydroxy-tetrahydrodipicolinate synthase, which is a key enzyme for lysine biosynthesis (Table 7). These proteins were not found among the down-expressed proteins in *E. faecium* (Suppl. Excel file 1), which supports the assumption that the bile acid stress response is unique in both organisms.

## Discussion

Tolerance against bile acid stress and microaerophilic conditions are key factors for pathogens and commensals to colonize the intestinal or the biliary tract.

The most abundant bile acid, CA, which is the precursor for secondary bile acids, is synthesized by the liver from cholesterol. CA has a steroid structure with three hydroxyl groups and a carboxyl group and it has a hydrocarbon side chain. The other primary bile acid CDCA differs from CA in its structure, it lacks one hydroxyl group. DCA, which is synthesized from CA has only one hydroxyl group [48].

We systematically investigated in this study adaptation processes that occur in *E. faecalis* and *E. faecium* after exposure to the three major bile acids in the human intestinal tract with a quantitative proteomic approach and correlated the obtained data with the inhibitory potential of these bile acids on bacterial growth.

### Similarities and differences in the bile acid adaptation processes between *E. faecalis* and *E. faecium*

Both microbial species displayed comparable susceptibility in their replication rate towards DCA and CDCA with an IC_50_ in the range of 0.01- 0.023%. Although the effect of the three bile acids on the growth phenotype is similar in both species, DIA-MS revealed differences in the proteomic response between the two *Enterococcus* species. Most strikingly, DCA and CDCA at 0.05% had a strong effect on down-expression of proteins assigned to the COG categories translation (J), transcription (K), and replication (L) in *E. faecalis*, resulting in a down-expression of 50% of the proteins assigned to these categories. A down-expression of 50% of the proteins in these categories indicates an effect on the fundamental functions of the COG categories and a particularly high stress level, which brings the cells to their adaptation limits. Due to the reduced growth with 0.05% of DCA and CDCA, the down-expression of these categories is not surprising. A linear relationship between growth phenotype and abundance of ribosomal proteins has been studied in *E. coli* and in other bacteria before [49]. This effect might also be present in *Enterococci.* Furthermore, the substantial reduction of growth might mask the stress response towards DCA and CDCA. However, this was not the case with CA.

Apparently, with CA, the stress level in both organisms was not as high as in DCA and CDCA, as the COG-categories translation (J), transcription (K), and replication (L) were not as much down-expressed. The down-expression level was between 10% and 25% of all proteins assigned to these categories.

On the other hand, in the approach of *E. faecalis* with CA, the COG-category J (translation) was highly up-expressed, as more than 50% of the identified proteins assigned to this category were significantly up-expressed (Fig. 2). It is worth to mention at this place that we determined the long-term effects of bile acids after 24 h exposure, while in many other transcriptomic or proteomic studies changes at much shorter time periods were analyzed [28–30]. The number of down-expressed proteins associated with translation, transcription, and replication was increased only moderately in *E. faecium*, between 20% and 40% of the identified proteins associated with these categories, suggesting a higher robustness to long term DCA and CDCA exposure.

The COG categories cell wall biogenesis (M) and chaperone production (O) were significantly up-expressed in both organisms (between 30% and 40% of the proteins assigned to categories M and O), when exposed to DCA and CDCA (Fig. 2), indicating that the maintenance and regeneration of the cell wall, the membrane and the protection of proteins via chaperones are of high importance under bile acid stress. Previous studies showed that bile acids disrupt the bacterial cell membrane [10, 12, 13, 50], thus, the proteomic response of the Enterococci fits to these findings. In the approach of *E. faecium* with exposure to CA, these COG-categories were not as much regulated as in DCA and CDCA, only between 0% and 10% of the proteins belonging to these categories were among the up-expressed proteins. This suggests that CA does not have the same impact on the cells.

Our results indicate that both *E. faecalis* and *E. faecium* can tolerate higher concentrations (more than 0.05%) of the primary bile acid CA compared to the secondary bile acids DCA and CDCA. Specifically, the growth phenotype of both *Enterococcus* species was almost unaffected by 0.05% CA, which is the bile acid with the highest concentration in the human gallbladder and duodenum [10, 27].

## Proteins commonly altered in their expression – a general (but species-specific) bile acid stress response

Comparative analysis of the samples exposed to DCA, CDCA and CA identified a subset of 283 commonly regulated proteins in *E. faecalis* and of 236 commonly regulated proteins in *E. faecium*. These proteins define the general stress response towards bile acids and thus are particularly useful for the identification of shared strategies by both species, but also allow the identification of species-specific mechanisms. A subset of 71 up-expressed proteins is shared at a concentration of 0.05% DCA, CDCA and CA in *E. faecalis*. Of these, 37 proteins are also up-expressed at a lower concentration of 0.01% DCA.

### V-type ATP-synthases

Among these shared up-expressed proteins were four subunits of a V-type ATPase, namely the ATP synthase alpha and beta chain, which form the catalytic hexamer [51–54], the subunit C, which is responsible for control of the assembly of the V-type ATPase [55], the subunits E and G, which are playing a role in the assembly of the ATPase and function as stalk [51], and the subunits D and I, whose exact function remains still unknown. In total, nine V-type ATPase subunits are present in the genome of *E. faecalis*, and we were able to identify all of them by DIA-MS. In *E. faecium*, eight V-type ATPase subunits are currently known. We found all eight by mass spectrometry.

V-type ATPases are membrane-bound proteins that are actively pumping ions, usually H^+^, out of the cell using ATP [53, 54]. These proton gradients are highly conserved in nature and have been shown to be crucial for survival in bile acid mediated stress before [28, 56]. This function has also been shown in *Lactobacillus plantarum* and *Bifidobacterium* sp [17, 57, 58]. The maintenance of a proton motive force in presence of bile also plays a role in other organisms. In *E. coli*, it has been shown that a bile acid secretion system might be driven by a proton motive force [59]. Furthermore, it has been demonstrated that a V-type ATPase is involved in regulating the intracellular Na^+^ concentration in the microbial species *Enterococcus hirae*. This ATPase facilitates an H^+^/Na^+^ antiport across the plasma membrane. The expression of this V-type ATPase is regulated at the transcriptional level by intracellular Na^+^ as an induction signal. Under acidic pH conditions, the H^+^ electrochemical gradient is generated by the H^+^-ATPase. This gradient drives the efflux of Na^+^ through the Na^+^/H^+^ antiporter and the influx of K^+^ via the KtrI transport system [60]. This might also be the case in *E. faecalis* and *E. faecium.* However, it is reasonable to assume that the V-type ATPase also contributes to an ion motive force that in turn can energize other plasma membrane transporters, which might be important to transport bile acids out of the cell.

In both organisms, the up-expression of V-type ATPase subunits was observed, however, the up-expression is seen only at a moderate level in *E. faecium.* From the eight detected V-type ATPase subunits in *E. faecium*, only one was up-expressed in all bile acids. This indicates that the contribution of V-type ATPase to the bile acid induced stress response might be slightly different for *E. faecium* and *E. faecalis.*

### ABC transporters

Several ABC transporter-related proteins as well as multidrug efflux proteins were found in the group of commonly up-expressed proteins in both, *E. faecalis* and *E. faecium.* These proteins might be relevant to actively transport bile acids out of the cell. This seems to be a similarity between both species but also fits to the observations in other species, such as *E. coli*, *Bifidobacterium longum* or *Campylobacter jejuni*, where bile acids are exported from the cell [57, 59, 61]. The up-expression of different transporters in both species as a response to bile acid exposition indicates that the process of transporting bile acids out of the cell is a conserved mechanism between bacteria.

The connection between antimicrobial resistance mechanisms and bile acid resistance mechanisms has been observed before, which explains the up-expression of the multidrug efflux pump proteins [62]. In 2017, Wulkersdorfer et al. showed that the efficacy of antibiotics decreases in the presence of bile acids in *E. faecalis* and *E. coli* [63]. Thus, it is likely, that the ABC transporters and multidrug resistance transporters we found to be up-expressed in *E. faecalis* and *E. faecium* are not only playing a role in antimicrobial resistance but also in bile resistance.

### Cell-wall biogenesis related proteins and metabolism

Proteins involved in peptidoglycan metabolism and murein synthesis were commonly up-expressed in all *E. faecalis* and *E. faecium* samples with bile acids. As bile acids disrupt the bacterial cell wall and membrane [11, 12, 26, 70], the synthesis of peptidoglycan and murein is thus a compensatory response to bile acid stress. This indicates that the integrity and maintenance of the bacterial cell wall plays an important role in adaptation to bile acids in both species. In contrast, down-expression of proteins involved in pyruvate-, citrate- and folate metabolism, e.g. the dihydrolipoamide acetyltransferase component BkdC of the pyruvate dehydrogenase complex (Q834J2), the citrate ligase CitC (Q82YW0), the dihydrofolate reductase FolA (Q834R2), or the 5-formyltetrahydrofolate cyclo-ligase (Q830J1), was only observed in *E. faecalis*, but not in *E. faecium*.

Together, our analysis of the proteomic response indicates similarities, but also significant differences in the adaptation towards bile acid stress in *E. faecalis* and *E. faecium*, even though these species are closely related [64, 65]. Whether these differences are adaptations to different microenvironments in the intestinal tract is currently unclear.

### Adaptation to the microaerophilic environment

*E. faecalis* usually inhabits the human gut, where the oxygen concentration is 1–2%. However, fecal-oral transmission is a common route for enterococcal infections, especially in infants. Due to its facultative anaerobic nature, *E. faecalis* is able to survive in normal oxygen conditions as well as in microaerophilic or anaerobic environments.

In fact, our growth comparison revealed a higher OD_600_ in microaerophilic environment than under aerobic conditions for *E. faecalis*. This suggests that *E. faecalis* is well adapted to a low oxygen atmosphere, which was also found in previous studies [66–68]. We found several ribosomal proteins among the up-expressed proteins under microaerophilic conditions, which suggests increased protein synthesis under these conditions. In samples with aerobic conditions, proteins involved in glycolysis and carbohydrate catabolism were upregulated when compared to microaerophilic samples. Among these proteins were a glyceraldehyde-3-phosphate dehydrogenase and components of the pyruvate dehydrogenase complex. This supports the observations of Portela et al. in 2014, who described an enhanced glycolysis metabolism of *E. faecalis* in an aerobic environment [69]. Most of the microaerophilic adaptations were also observed in the presence of DCA. This indicates that DCA has a strong influence on the bacteria in an aerobic as well as in microaerophilic atmosphere but does not prevent the microaerophilic proteomic response.

### Electronic supplementary material

Below is the link to the electronic supplementary material.

Supplementary Material 1

Supplementary Material 2

Supplementary Material 3

## Data Availability

Data are available via ProteomeXchange with identifier PXD040819. Submission details: Project Name: comparative analysis of proteomic adaptations in *Enterococcus faecalis* and *Enterococcus faecium* after long term bile acid exposure. Project accession: PXD040819. Project DOI: 10.1186/s12866-024-03253-0. Reviewer account details: Username: reviewer_pxd040819@ebi.ac.uk Password: tSZJmLHN.
