# Supplementary material for: Comparative analysis of proteomic adaptations in Enterococcus faecalis and Enterococcus faecium after long term bile acid exposure
Source: BMC Microbiol. 2024 Apr 3;24:110. doi: 10.1186/s12866-024-03253-0 (PMC10988882; doi:10.1186/s12866-024-03253-0)

**Supplementary Tables and Figures**

**Supp.-Table 1:** 72 commonly significantly up-expressed proteins in E. faecalis samples with 0.05 % DCA, CDCA or CA. Proteins involved in murein or peptidoglycan synthesis are marked in green, transporter proteins are marked in blue and V-type ATPases are marked in yellow.

| ‍**Uniprot ID** | **Protein function** |
| --- | --- |
| VATB_ENTFA | V-type ATP synthase beta chain |
| VATA_ENTFA | V-type ATP synthase alpha chain |
| Q834Y2_ENTFA | V-type ATPase, subunit E |
| Q834Y4_ENTFA | V-type ATP synthase subunit I |
| H7C6V7_ENTFA | penicillin-binding protein 4 |
| H7C713_ENTFA | cell division protein DivIVA |
| H7C714_ENTFA | glucose-1-phosphate thymidylyltransferase |
| H7C7A3_ENTFA | PTS system, IIB component |
| MSRB_ENTFA | peptide methionine sulfoxide reductase MsrB |
| Q820A8_ENTFA | non-specific serine/threonine protein kinase |
| Q820U5_ENTFA | SPFH domain/Band 7 family protein |
| Q82YY5_ENTFA | ribosome-binding ATPase YchF |
| Q82YZ9_ENTFA | peptidase, U32 family, putative |
| Q82ZA8_ENTFA | hydrolase, haloacid dehalogenase-like family |
| Q82ZH5_ENTFA | iron compound ABC transporter, substrate-binding protein |
| Q82ZT6_ENTFA | ribokinase |
| Q82ZY6_ENTFA | DNA-binding response regulator, LuxR family |
| Q82ZY8_ENTFA | uncharacterized protein |
| Q82ZZ9_ENTFA | peptidyl-prolyl cis-trans isomerase |
| Y2866_ENTFA | probable transcriptional regulatory protein EF_2866 |
| Q830E0_ENTFA | uncharacterized protein |
| Q830N7_ENTFA | lipoate--protein ligase |
| RL7_ENTFA | 50S ribosomal protein L7/L12 |
| Q830X4_ENTFA | diacylglycerol kinase catalytic domain protein |
| ATPF_ENTFA | ATP synthase subunit b |
| ATPB_ENTFA | ATP synthase subunit beta |
| Q831B8_ENTFA | ABC transporter, ATP-binding/permease protein |
| Q831B9_ENTFA | ABC transporter, ATP-binding/permease protein |
| RF1_ENTFA | peptide chain release factor 1 |
| Q831F8_ENTFA | threonylcarbamoyl-AMP synthase |
| ISPT_ENTFA | isoprenyl transferase |
| Q831R2_ENTFA | PTS system, IIA component |
| ACYP_ENTFA | acylphosphatase |
| EFTS_ENTFA | elongation factor Ts |
| Q831Z9_ENTFA | uncharacterized protein |
| Q832A0_ENTFA | uncharacterized protein |
| Q832N1_ENTFA | dTDP-glucose 4,6-dehydratase |
| Q833B2_ENTFA | oxidoreductase, pyridine nucleotide-disulfide family |
| MURC_ENTFA | UDP-N-acetylmuramate--L-alanine ligase |
| Q834B0_ENTFA | phosphate-binding protein |
| Q834B6_ENTFA | DUF4097 domain-containing protein |
| Q834C4_ENTFA | general stress protein, putative |
| Q834G9_ENTFA | DegV family protein, putative |
| Q834H3_ENTFA | carboxyl-terminal protease |
| Q834K8_ENTFA | iron compound ABC transporter, iron compound-binding protein |
| Q834T0_ENTFA | TPR domain protein |
| Q834T5_ENTFA | ribosomal protein S1 |
| Q834U8_ENTFA | peptidyl-prolyl cis-trans isomerase |
| Q835H5_ENTFA | uncharacterized protein |
| Q835L0_ENTFA | cold-shock domain family protein |
| Q835Q5_ENTFA | ABC transporter, ATP-binding protein |
| DNAK_ENTFA | chaperone protein DnaK |
| GRPE_ENTFA | protein GrpE |
| Q835V8_ENTFA | sulfatase domain protein |
| Y1202_ENTFA | UPF0297 protein EF_1202 |
| MURA1_ENTFA | UDP-N-acetylglucosamine 1-carboxyvinyltransferase 1 |
| Q836G8_ENTFA | lipoyl-[GcvH]:protein N-lipoyltransferase |
| Q837D6_ENTFA | peptide ABC transporter, peptide-binding protein |
| Q837E6_ENTFA | preprotein translocase, YajC subunit, putative |
| QUEA_ENTFA | S-adenosylmethionine:tRNA ribosyltransferase-isomerase |
| Q837J3_ENTFA | UDP-N-acetylmuramoyl-tripeptide--D-alanyl-D-alanine ligase |
| TIG_ENTFA | trigger factor |
| PRSA_ENTFA | foldase protein PrsA |
| Q838H8_ENTFA | ribonucleoside-diphosphate reductase subunit beta |
| Q838M3_ENTFA | transcriptional regulator, MerR family |
| Q838M4_ENTFA | drug resistance transporter, EmrB/QacA family protein |
| Q838M5_ENTFA | uncharacterized protein |
| Q838Q5_ENTFA | abhydrolase_3 domain-containing protein |
| EFP_ENTFA | elongation factor P |
| EFTU_ENTFA | elongation factor Tu |
| Q839I8_ENTFA | basic membrane protein family |

**Supp.-Table 2:** 40 commonly significantly up-expressed proteins in E. faecalis samples with 0.05 % DCA, CDCA or CA and 0.01 % DCA in aerobic conditions. Proteins involved in murein or peptidoglycan synthesis are marked in green, transporter proteins are marked in blue and V-type ATPases are marked in yellow.

| ‍**Uniprot ID** | **Protein function** |
| --- | --- |
| H7C6V7_ENTFA | penicillin-binding protein 4 |
| H7C713_ENTFA | cell division protein DivIVA |
| H7C714_ENTFA | glucose-1-phosphate thymidylyltransferase |
| Q820A8_ENTFA | non-specific serine/threonine protein kinase |
| Q82YZ9_ENTFA | peptidase, U32 family, putative |
| Q82ZA8_ENTFA | hydrolase, haloacid dehalogenase-like family |
| Q82ZH5_ENTFA | iron compound ABC transporter, substrate-binding protein |
| Y2866_ENTFA | probable transcriptional regulatory protein EF_2866 |
| Q830N7_ENTFA | lipoate--protein ligase |
| Q830X4_ENTFA | diacylglycerol kinase catalytic domain protein |
| ATPF_ENTFA | ATP synthase subunit b |
| Q831B8_ENTFA | ABC transporter, ATP-binding/permease protein |
| Q831B9_ENTFA | ABC transporter, ATP-binding/permease protein |
| RF1_ENTFA | peptide chain release factor 1 |
| Q831R2_ENTFA | PTS system, IIA component |
| EFTS_ENTFA | elongation factor Ts |
| Q832A0_ENTFA | uncharacterized protein |
| Q832N1_ENTFA | dTDP-glucose 4,6-dehydratase |
| Q833B2_ENTFA | oxidoreductase, pyridine nucleotide-disulfide family |
| MURC_ENTFA | UDP-*N*-acetylmuramate--L-alanine ligase |
| Q834B6_ENTFA | DUF4097 domain-containing protein |
| Q834G9_ENTFA | DegV family protein, putative |
| Q834T0_ENTFA | TPR domain protein |
| VATB_ENTFA | V-type ATP synthase beta chain |
| VATA_ENTFA | V-type ATP synthase alpha chain |
| Q834Y2_ENTFA | V-type ATPase, subunit E |
| Q834Y4_ENTFA | V-type ATP synthase subunit I |
| DNAK_ENTFA | chaperone protein DnaK |
| GRPE_ENTFA | protein GrpE |
| Q835V8_ENTFA | sulfatase domain protein |
| MURA1_ENTFA | UDP-*N*-acetylglucosamine 1-carboxyvinyltransferase 1 |
| QUEA_ENTFA | S-adenosylmethionine:tRNA ribosyltransferase-isomerase |
| Q837J3_ENTFA | UDP-*N*-acetylmuramoyl-tripeptide--D-alanyl-D-alanine ligase |
| TIG_ENTFA | trigger factor |
| Q838M3_ENTFA | transcriptional regulator, MerR family |
| Q838M4_ENTFA | drug resistance transporter, EmrB/QacA family protein |
| Q838M5_ENTFA | uncharacterized protein |
| Q838Q5_ENTFA | abhydrolase_3 domain-containing protein |
| EFP_ENTFA | elongation factor P |
| EFTU_ENTFA | elongation factor Tu |

**Supp.-Table 3:** 424 commonly down-expressed proteins in E. faecalis samples with 0.05 % DCA, CDCA, CA.

| ‍**Uniprot ID** | **Protein function** |
| --- | --- |
| H7C6V1_ENTFA | AAA domain-containing protein |
| H7C6X0_ENTFA | transcriptional regulator, TetR family |
| H7C6X1_ENTFA | prephenate dehydrogenase |
| H7C6X2_ENTFA | prephenate dehydratase |
| H7C6Z2_ENTFA | PrgU domain-containing protein |
| H7C6Z5_ENTFA | 2-dehydropantoate 2-reductase |
| H7C710_ENTFA | branched-chain alpha-keto acid dehydrogenase, E1 component, beta subunit |
| H7C717_ENTFA | xylose repressor, putative |
| H7C718_ENTFA | single-stranded DNA-binding protein |
| PARE_ENTFA | DNA topoisomerase 4 subunit B |
| H7C798_ENTFA | transcriptional regulator, putative |
| H7C7A4_ENTFA | arginine repressor |
| H7C7B6_ENTFA | transcriptional regulator, TetR family |
| H7C7C0_ENTFA | uncharacterized protein |
| Q837H9_ENTFA | APC family amino acid-polyamine-organocation transporter, membrane protein, putative |
| AROA_ENTFA | 3-phosphoshikimate 1-carboxyvinyltransferase |
| AROB_ENTFA | 3-dehydroquinate synthase |
| AROD_ENTFA | 3-dehydroquinate dehydratase |
| Q833S0_ENTFA | ABC superfamily ATP binding cassette transporter, ABC/membrane protein, ABC transporter, ATP-binding protein |
| MIAA_ENTFA | tRNA dimethylallyltransferase |
| Q820V7_ENTFA | transcriptional regulator, Crp/Fnr family |
| Q82YI2_ENTFA | replication control protein PrgN |
| Q82YI6_ENTFA | DUF262 domain-containing protein |
| Q82YI7_ENTFA | site-specific recombinase, resolvase family |
| Q82YK3_ENTFA | uncharacterized protein |
| Q82YN8_ENTFA | pheromone shutdown protein TraB |
| Q82YP1_ENTFA | replication-associated protein RepB |
| Q82YQ0_ENTFA | site-specific recombinase, resolvase family, site-specific recombinase, resolvase family |
| Q82YR3_ENTFA | sucrose operon repressor ScrR |
| Q82YT1_ENTFA | uncharacterized protein |
| Q82YT5_ENTFA | AAA domain-containing protein |
| Q82YT9_ENTFA | uncharacterized protein |
| Q82YV4_ENTFA | transcriptional regulator, GntR family |
| Q82YW0_ENTFA | citrate pro-3S-lyase ligase |
| CITD_ENTFA | citrate lyase acyl carrier protein |
| RSMG_ENTFA | ribosomal RNA small subunit methyltransferase G |
| Q82YZ7_ENTFA | ATP-dependent Clp protease, ATP-binding subunit ClpC |
| Q82Z36_ENTFA | abortive phage resistance protein, putative |
| RPOC_ENTFA | DNA-directed RNA polymerase subunit beta |
| Q82Z57_ENTFA | helicase, putative |
| Q82Z79_ENTFA | isochorismatase family protein |
| Q82ZA3_ENTFA | DNA mismatch repair protein MutL |
| KPRS2_ENTFA | ribose-phosphate pyrophosphokinase 2 |
| Q82ZC1_ENTFA | phosphosugar-binding transcriptional regulator, RpiR family |
| Q82ZC2_ENTFA | 6-phosphogluconate dehydrogenase family protein |
| UXUA_ENTFA | mannonate dehydratase |
| Q82ZD3_ENTFA | uncharacterized protein |
| Q82ZD7_ENTFA | primosomal protein N |
| Q82ZE3_ENTFA | thiamine pyrophosphokinase family protein |
| Q82ZF0_ENTFA | peptide ABC transporter, ATP-binding protein |
| Q82ZF1_ENTFA | peptide ABC transporter, ATP-binding protein |
| Q82ZF2_ENTFA | peptide ABC transporter, permease protein |
| Q82ZK6_ENTFA | phosphosugar-binding transcriptional regulator, RpiR family, putative |
| Q82ZN7_ENTFA | Mga domain-containing protein |
| Q82ZP4_ENTFA | uncharacterized protein |
| Q82ZQ4_ENTFA | uncharacterized protein |
| Q82ZQ5_ENTFA | aminotransferase, class V |
| Q82ZS2_ENTFA | pyridoxal phosphate-dependent enzyme, putative, pyridoxal phosphate-dependent enzyme, putative |
| Q82ZS3_ENTFA | uncharacterized protein |
| Q82ZT5_ENTFA | sugar-binding transcriptional regulator, LacI family |
| Q82ZT9_ENTFA | transcriptional regulator, LysR family |
| REX2_ENTFA | redox-sensing transcriptional repressor Rex 2 |
| Q830A9_ENTFA | transcriptional regulator, MarR family |
| ACCD_ENTFA | acetyl-coenzyme A carboxylase carboxyl transferase subunit beta |
| Q830J1_ENTFA | 5-formyltetrahydrofolate cyclo-ligase |
| Q830J4_ENTFA | glucokinase |
| Q830J7_ENTFA | NAD binding 9 domain-containing protein |
| Q830K1_ENTFA | DNA polymerase III subunit gamma/tau |
| Q830L8_ENTFA | DNA polymerase III, delta prime subunit |
| Q830N9_ENTFA | thioredoxin reductase/glutathione-related protein |
| Q830S2_ENTFA | MutT/nudix family protein |
| Q830T4_ENTFA | Snf2 family protein |
| Q830T5_ENTFA | uncharacterized protein |
| Q830X2_ENTFA | glycerate kinase, putative |
| Q830X5_ENTFA | uncharacterized protein |
| Q831D4_ENTFA | aspartate/ornithine carbamoyltransferase family protein |
| Q831L3_ENTFA | glycosyl transferase, group 2 family protein |
| Q831L5_ENTFA | UDP-*N*-acetylenolpyruvoylglucosamine reductase |
| Q831L7_ENTFA | UDP-galactopyranose mutase |
| TSAD_ENTFA | tRNA N6-adenosine threonylcarbamoyltransferase |
| Q831R6_ENTFA | phosphosugar-binding transcriptional regulator, RpiR family |
| Q831S4_ENTFA | transcriptional regulator, GntR family |
| Q831S7_ENTFA | transcriptional regulator, ArsR family |
| Q831W1_ENTFA | uncharacterized protein |
| LEPA_ENTFA | elongation factor 4 |
| Q832J0_ENTFA | uncharacterized protein |
| Q832J1_ENTFA | glucuronyl hydrolase, putative |
| Q832L4_ENTFA | uncharacterized protein |
| Q832L6_ENTFA | uncharacterized protein |
| Q832L9_ENTFA | DNA-binding protein, Fis family |
| Q832M2_ENTFA | transcriptional regulator, TetR family |
| Q832P8_ENTFA | 2-C-methyl-D-erythritol 4-phosphate cytidylyltransferase |
| Q832Q4_ENTFA | membrane protein, putative |
| Q832Q8_ENTFA | GTPase HflX |
| Q832Y2_ENTFA | endolysin |
| Q833B0_ENTFA | heptaprenyl diphosphate synthase, component II, putative |
| Q833B1_ENTFA | 1,4-dihydroxy-2-naphthoate octaprenyltransferase, putative |
| Q833I3_ENTFA | phosphomethylpyrimidine kinase, putative |
| Q833L4_ENTFA | uncharacterized protein |
| Q833L5_ENTFA | oxidoreductase, pyridine nucleotide-disulfide family |
| Q833L7_ENTFA | alpha-glycerophosphate oxidase |
| Q833M2_ENTFA | ribokinase |
| Q833N2_ENTFA | ROK family protein |
| Q833P3_ENTFA | divalent metal cation transporter MntH |
| LACA_ENTFA | galactose-6-phosphate isomerase subunit LacA |
| LACB_ENTFA | galactose-6-phosphate isomerase subunit LacB |
| Q833V0_ENTFA | alcohol dehydrogenase, zinc-containing |
| Q833X6_ENTFA | uncharacterized protein |
| Q834B2_ENTFA | phosphate transport system permease protein PstA |
| Q834C8_ENTFA | uncharacterized protein |
| Q834E5_ENTFA | transcriptional regulator, LysR family |
| Q834F2_ENTFA | alkaline phosphatase synthesis transcriptional regulatory protein PhoP |
| Q834F5_ENTFA | signal recognition particle protein |
| Q834I2_ENTFA | phospholipase/carboxylesterase family protein |
| Q834I9_ENTFA | branched-chain phosphotransacylase |
| Q834J0_ENTFA | dihydrolipoyl dehydrogenase |
| Q834J1_ENTFA | branched-chain alpha-keto acid dehydrogenase, E1 component, alpha subunit |
| Q834J2_ENTFA | dihydrolipoamide acetyltransferase component of pyruvate dehydrogenase complex |
| Q834J3_ENTFA | membrane protein, putative |
| Q834J4_ENTFA | transcriptional regulator, LysR family |
| Q834J5_ENTFA | 2-dehydropantoate 2-reductase |
| Q834N0_ENTFA | DNA topoisomerase 4 subunit A |
| Q834N6_ENTFA | cardiolipin synthase |
| Q834N9_ENTFA | sucrose operon repressor ScrR |
| Q834R2_ENTFA | dihydrofolate reductase |
| Q834U7_ENTFA | uncharacterized protein |
| Q834W2_ENTFA | PTS system, IIABC components |
| Q834X2_ENTFA | Hydrolase 4 domain-containing protein |
| Q834Y9_ENTFA | uncharacterized protein |
| Q835H7_ENTFA | cadmium-translocating P-type ATPase |
| Q835K5_ENTFA | CBS domain protein |
| Q835V0_ENTFA | YlxR domain-containing protein |
| RIMP_ENTFA | ribosome maturation factor RimP |
| Q835X6_ENTFA | transcriptional regulator, GntR family |
| Q835Y1_ENTFA | endo/exonuclease/phosphatase domain-containing protein |
| Q835Z1_ENTFA | FMN red domain-containing protein |
| Q836C5_ENTFA | uncharacterized protein |
| DAPA_ENTFA | 4-hydroxy-tetrahydrodipicolinate synthase |
| Q836F5_ENTFA | PTS system, cellobiose-specific IIB component |
| Q836F8_ENTFA | endonuclease III |
| Q836H0_ENTFA | hydrolase, haloacid dehalogenase-like family |
| Q836J7_ENTFA | HTH hxlR-type domain-containing protein |
| ADDA_ENTFA | ATP-dependent helicase/nuclease subunit A |
| ADDB_ENTFA | ATP-dependent helicase/deoxyribonuclease subunit B |
| Q836M6_ENTFA | universal stress protein |
| Q836N7_ENTFA | galactose operon repressor galR |
| Q836Q8_ENTFA | DNA-binding response regulator |
| Q836S2_ENTFA | nucleoside diphosphate kinase |
| Q836S5_ENTFA | 6-aminohexanoate-cyclic-dimer hydrolase, putative |
| Q836S6_ENTFA | daunorubicin resistance protein |
| Q836T6_ENTFA | *N*-acetyltransferase domain-containing protein |
| Q836U0_ENTFA | PTS system, IIB component;PTS system, IIB component |
| Q836U3_ENTFA | uncharacterized protein |
| Q836U4_ENTFA | Permease IIC component |
| Q836V5_ENTFA | Pyridoxal phosphate homeostasis protein |
| Q836V8_ENTFA | lipoprotein, putative |
| Q836Y4_ENTFA | endonuclease/exonuclease/phosphatase family protein |
| Q837A3_ENTFA | uncharacterized protein |
| Q837C0_ENTFA | transcriptional regulator, LysR family |
| IF3_ENTFA | translation initiation factor IF-3 |
| IDI2_ENTFA | isopentenyl-diphosphate delta-isomerase |
| Q837E3_ENTFA | aldehyde-alcohol dehydrogenase |
| TGT_ENTFA | queuine tRNA-ribosyltransferase |
| Q837H3_ENTFA | glyoxalase family protein |
| Q837J7_ENTFA | carbohydrate kinase, pfkB family |
| Q837K3_ENTFA | permease IIC component |
| Q837L3_ENTFA | acetyltransferase, GNAT family |
| Q837M3_ENTFA | beta-galactosidase |
| Q837M4_ENTFA | glucuronyl hydrolase, putative |
| Q837P3_ENTFA | transcriptional regulator, TetR family |
| Q837P4_ENTFA | ABC transporter, ATP-binding/permease protein |
| Q837P7_ENTFA | tributyrin esterase, putative |
| METK_ENTFA | S-adenosylmethionine synthase |
| Q837Q1_ENTFA | RNA polymerase sigma-54 factor |
| Q837S3_ENTFA | cadmium-translocating P-type ATPase |
| Q837V7_ENTFA | ATP-dependent DNA helicase |
| Q837W5_ENTFA | WxL domain-containing protein |
| Q837Z0_ENTFA | Cmp-binding protein, putative |
| Q838B3_ENTFA | Type-4 uracil-DNA glycosylase |
| Q838D8_ENTFA | cardiolipin synthase |
| Q838D9_ENTFA | glyoxalase family protein |
| Q838E0_ENTFA | oxidoreductase, aldo/keto reductase family |
| Q838Q3_ENTFA | uncharacterized protein |
| Q838Z1_ENTFA | glycosyl hydrolase, family 1 |
| Q838Z2_ENTFA | cystathionine beta-lyase |
| Q839A9_ENTFA | tRNA-dihydrouridine synthase |
| Q839B7_ENTFA | S4 RNA-binding domain protein |
| Q839B9_ENTFA | transcription-repair-coupling factor |
| Q839C2_ENTFA | aldehyde dehydrogenase |
| Q839C4_ENTFA | transcriptional regulator, putative |
| RS3_ENTFA | 30S ribosomal protein S3 |
| Q839G7_ENTFA | phosphomethylpyrimidine kinase, putative |
| Q839P5_ENTFA | transcriptional regulator, GntR family |
| Q839Q7_ENTFA | hydrolase, alpha/beta hydrolase fold family |
| SYS1_ENTFA | serine--tRNA ligase 1 |
| Q839Q9_ENTFA | L-serine dehydratase |
| Q839R2_ENTFA | uncharacterized protein |
| Q839T0_ENTFA | oxidoreductase, short chain dehydrogenase/reductase family |
| Q839U2_ENTFA | pur operon repressor |
| SYC_ENTFA | cysteine--tRNA ligase |
| ISPF_ENTFA | 2-C-methyl-D-erythritol 2,4-cyclodiphosphate synthase |
| Q839W5_ENTFA | uncharacterized protein |
| Q839Y1_ENTFA | Sigma-54 interaction domain protein |
| Q839Z3_ENTFA | S4 RNA-binding domain-containing protein |
| BUK_ENTFA | probable butyrate kinase |
| Q82YT2_ENTFA | AAA domain-containing protein |
| Q82YS4_ENTFA | PrgU domain-containing protein |
| Q82YR0_ENTFA | site-specific recombinase, resolvase family |
| Q838G7_ENTFA | conjugal transfer protein |
| Q837J9_ENTFA | pyridoxal phosphate-dependent enzyme, putative |
| Q837J8_ENTFA | oxo-acid lyase |
| Q836U5_ENTFA | PTS system, IIB component |

**Supp.-Figure 1:** Doughnut diagram of the percentage distribution of the COG categories of the common regulated proteins in *E. faecalis* (A) and *E. faecium* (B). In *E. faecalis*, 323 proteins were significantly differentially expressed in common in presence of 0.05 % DCA, CDCA and CA, in *E. faecium*, 246 proteins are regulated commonly. The significant similarities between the bile salt stress proteomes suggest a strong conservation between the two species.


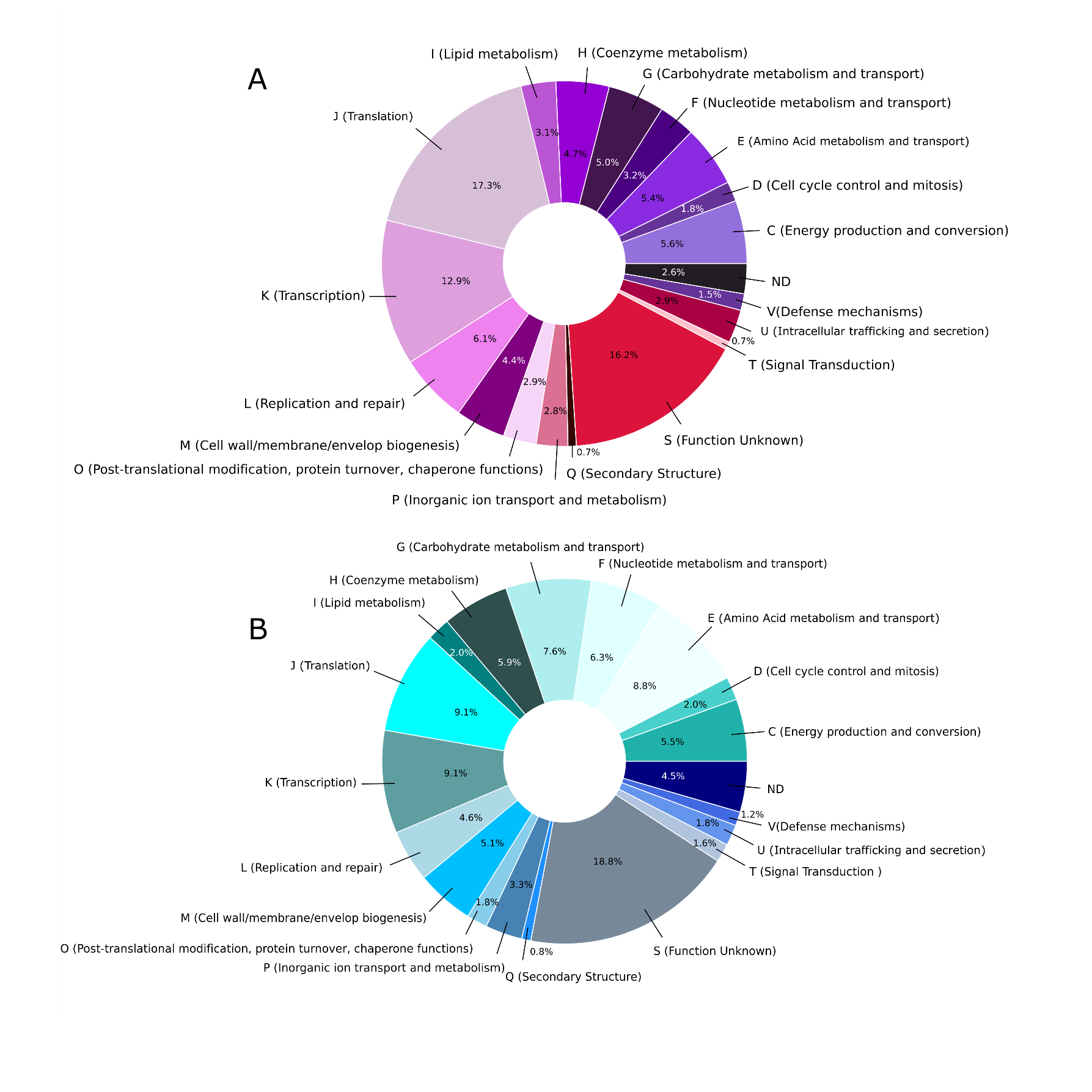


**Supp.-Figure 2:** Venn diagram of all significantly differentially expressed proteins in the approach of *E. faecalis* in microaerophilic conditions (A) and microaerophilic conditions with 0.01 % DCA (B), compared to aerobic conditions, respectively.


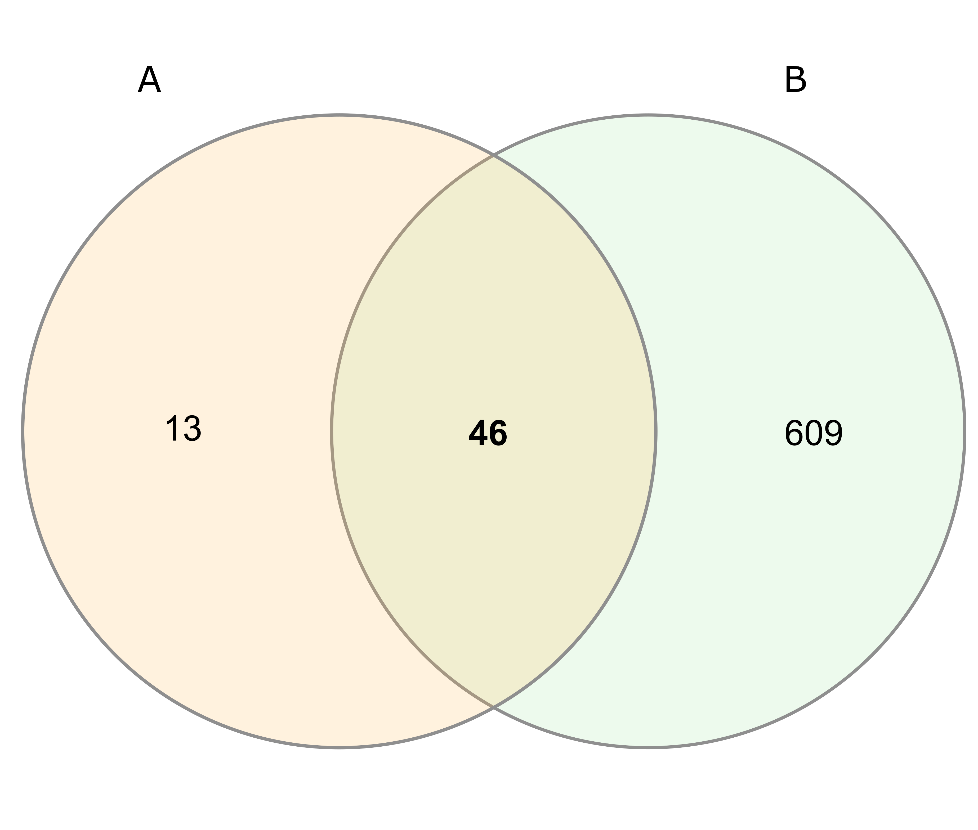


**Supp.-Figure 3:** Venn diagrams of proteins that were significantly up-expressed in all approaches with all bile salts and additionally with 0.01 % DCA in oxic (**oxy**) and microaerophilic (**micr**) conditions in *E. faecalis*. 37 proteins were commonly up-expressed in all approaches.


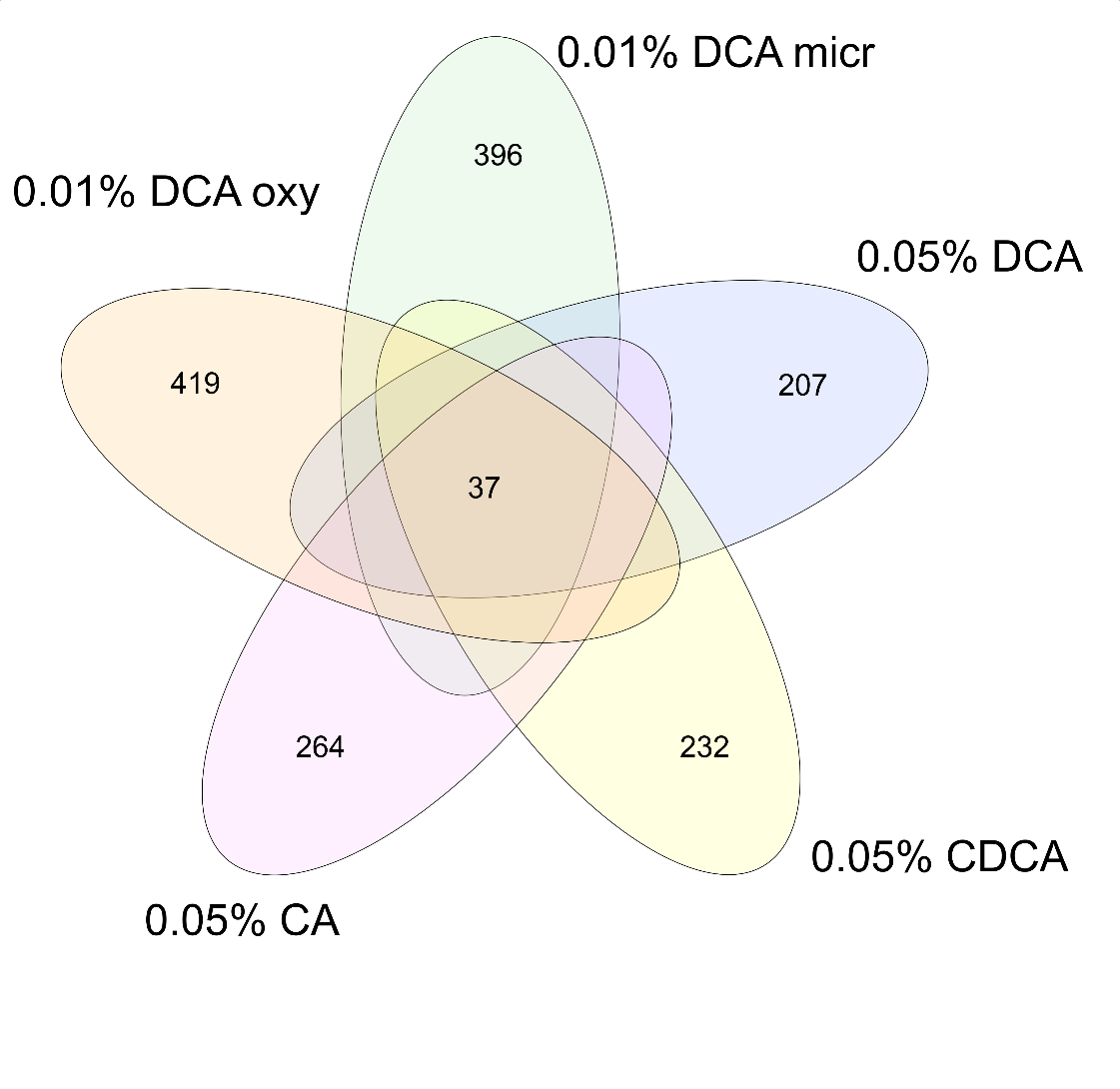

Supplement: Supplementary file 1 — Supplementary Material 1 [file 12866_2024_3253_MOESM1_ESM.docx]
